# Supplementary material for: Sustainable development goals and multisectoral collaborations for child health in Cambodia: a qualitative interview study with key child health stakeholders
Source: BMJ Open. 2023 Nov 21;13(11):e073853. doi: 10.1136/bmjopen-2023-073853 (PMC10668300; doi:10.1136/bmjopen-2023-073853)
Supplement: Supplementary data [file bmjopen-2023-073853supp001.pdf]

Supplementary Material 1

Table of Contents

Interview Guide 2

Consolidated criteria for reporting qualitative studies (COREQ): 32-item checklist 6

Full coding tables 9

Examples of multisectoral collaboration that include child health and well-being in Cambodia 21

Helldén D, *et al. BMJ Open* 2023; 13:e073853. doi: 10.1136/bmjopen-2023-073853

Interview Guide

1. Background information. *Please mark and fill in the following questions on this sheet*

**1. How many years have you worked:** ________
**2. Sex** ☐ Female ☐ Male
**3. Education**<br><br>Highest degree:____<br><br>Topic of degree:____

**4. Work experience**					
**Current employment**<br><br>**Organization:**______________________________ **Role/Position:**________________________________					
**Work experience from the following sectors (represented by Cambodia Sustainable Development Goals) - Multiple Answers**					
**Sectors**	**Rural**	**Urban**	**Sectors**	**Rural**	**Urban**
1. Poverty/social protection	☐	☐	10. Income inequality	☐	☐
2. Food and nutrition	☐	☐	11. Sustainable cities/communities/urban planning	☐	☐
3. Health and well-being	☐	☐	12. Safe consumption and production	☐	☐
4. Education	☐	☐	13. Climate change	☐	☐
5. Gender equality	☐	☐	14. Life bellow water/ocean	☐	☐
6. Water and sanitation	☐	☐	15. Life on land/natural resources	☐	☐
7. Energy	☐	☐	16. Institutional strengthening/anti corruption/legislation	☐	☐
8. Labor market, financial sector	☐	☐	17. Partnerships/collaborative networks	☐	☐

Helldén D, *et al. BMJ Open* 2023; 13:e073853. doi: 10.1136/bmjopen-2023-073853

9. Industry and infrastructure	☐	☐	18. Cambodia Mine/ERW free	☐	☐
Other:				☐	☐
**Have you worked related to child health (both health and non-health sector)?** ☐ Yes ☐ No<br><br>**If yes, please describe shortly in what way:**					

Thank you for providing telling us your background, we would now like to know a little bit more about your views on the topics of Sustainable Development, child health and multisectoral work in general.

2. Background: Sustainable Development Goals, child health and multisectoral work

The 2030 Agenda and the 17 Sustainable Development Goals is a framework with a comprehensive set of goals adopted by the UN in 2015 for all countries to end poverty, protect the planet and ensure prosperity for all. These have been adapted to the Cambodia Sustainable Goals.

2.1. What were your first thoughts/opinion when the 2030 Agenda and the Sustainable Development Goals were launched?

2.1.2. What have your organization done now in relation to the Sustainable Development Goals in Cambodia compared to what you did with the Millennium Development Goals before and? (*mode of work rather than specific activities*)

2.2. Could you please describe what you would say child health includes?

2.2.1 What age? What to you include in the term "health" when it comes to children?

2.2.2 Are there any particular aspects of the health of children that you think the health system need to take special consideration to?

2.3. How do you think actors in Cambodia contribute in supporting child health to implement the Cambodia Sustainable Development Goals?

2.4. What are the linkages/connections between child health and non-health sectors (as represented by the Cambodia Sustainable Development Goals)?

2.4.1 Please provide examples of such linkages from your current/former work or what you have observe in the society?

2.4.2 Many sectors and activities can influence child health. Which sectors do you think are most relevant for child health?

Thank you for providing your views on these topics, now we would like to ask you some further questions on the multisectoral work around child health that you have experience from. Please think of a collaboration specifically, or generally if you have experience from many different, between <u>two or more sectors</u> that had the explicit goal to in some way increase child health and well-being.

<u>3. Multisectoral collaboration for child health</u>

Based on the Multisectoral collaborative model and the Health in all policies approach.

i) Drive change/Establishing the need for multisectoral work

3.1. Which organizations are your key stakeholders to work in promoting child health?

3.1. What was it that made the partners in the collaboration identify the need for multisectoral work? How did it begin?

ii) Defining the problem and constraints

3.2. How was the above-mentioned child health need identified, defined or framed?

iii) Design of the collaboration/Planned framed action & Supportive structures and policies

3.3. Was there a planning process of how to conduct the multisectoral work?

3.4. Which stakeholders were involved in planning?

3.5. How was the work of collaboration designed to be carried out?

3.5.1 How was the coordination organized?

3.5.2 How was the collaboration implemented?

3.5.3 How was the work of the collaboration financed or mobilized?

3.5. How do you think the multisectoral work were actually implemented compared to the plan?

3.6. Where there any supportive structures or policies in place that enabled the work to be conducted?

iv) Capture success / Monitoring and evaluation

3.7. How was the multisectoral work monitored and evaluated?

3.7.1 Was there any key indicators or markers of success monitored?

3.7.2 How was the success or failure attributed to between the partners in the collaboration?

V) Relate / Facilitate assessment and engagement & Build capacity

3.8. How did the relationship between the partners evolve during the multisectoral work?

3.9. Did the collaborating partners make any effort to improve their relationship?

3.10. Where there any efforts to engage with a wider group of actors or the public in the work?

4. Where there any type of capacity building activities included in the collaboration?

Final questions

5.1. What are your suggestions and recommendations in order to improve multisectoral collaboration to promote child health?

5.2. Are there any end points you want to add on any of the topics touched upon today or that we have not spoken about?

Helldén D, *et al. BMJ Open* 2023; 13:e073853. doi: 10.1136/bmjopen-2023-073853

Consolidated criteria for reporting qualitative studies (COREQ): 32-item checklist

No.	Item	Description	Page
Domain 1: Research team and reflexivity			
Personal characteristics			
1	Interviewer/facilitator	SS and TC conducted the interviews	4
2	Credentials	DH has a MD, SS has PhD, TC has MD and has a Master of Arts in Health Social Sciences, HN has a PhD, KR has a PhD, HMA has a PhD and TA has a PhD.	1
3	Occupation	DH was a PhD candidate, SS was a lecturer at the Royal University of Phnom Penh, TC was a program manager at Malaria Consortium Cambodia.	1
4	Gender	All interviewers were male.	NA
5	Experience and training	The researchers (SS and TC) had extensive experience of qualitative interviews from previous research in Cambodia.	16
Relationship with participants			
6	Relationship established	No relationship was established prior to study commencement.	16
7	Participant knowledge of the interviewer	In some instances, the participant recognized the interviewer from attending similar events/workshops/seminars but in general the participants did not know the interviewers. They did not know the personal goals or reasons for doing the research for the individual interviewer.	16
8	Interviewer characteristics	The interviewers were all interested in the topic in general and had expertise in child health in Cambodia.	4
Domain 2: Study design			
Theoretical framework			

Helldén D, et al. BMJ Open 2023; 13:e073853. doi: 10.1136/bmjopen-2023-073853

9	Methodological orientation and theory	The methodological orientation of the study is content analysis, specifically framework analysis.	4
Participant selection			
10	Sampling	Participants were purposively selected based on predefined criteria of having expertise in child health or being from a non-health sector (for example water and sanitation, agriculture, infrastructure etc.) but with implementation knowledge of how that sector interacts with other sectors in Cambodia.	4
11	Method of approach	Participants were approached via email and telephone.	NA
12	Sample size	29 participated in the interviews.	4
13	Non-participation	No participants refused or dropped out.	4
Setting			
14	Setting of data collection	The interviews took place either virtually (over online meeting) or face to face, at a time and place convenient of the participant.	4
15	Presence of non-participants	The were no non-participants present during the interviews.	4
16	Description of sample	The description of the sample can be seen in Table 1 in the article.	5
Data collection			
17	Interview guide	This is provided in the supplementary material 1. The interview guide was piloted before the study began.	4-5
18	Repeat inverviews	No repeat interviews were held.	NA
19	Audio/visual recording	Audio recording was used to collect the data.	4
20	Field notes	No field notes were taken.	4
21	Duration	The duration of the interviews ranged from 45 minutes to 1 hr and 15 minutes	NA
22	Data saturation	Is discussed with regards to information power in the article.	16

Helldén D, *et al. BMJ Open* 2023; 13:e073853. doi: 10.1136/bmjopen-2023-073853

23	Transcripts returned	Transcripts were not returned to participants.	NA
Domain 3: Analysis and findings			
Data analysis			
24	Number of data coders	DH coded the data	8
25	Description of the coding tree	Is presented in Table 2 in the manuscript and supplementary material 1.	9
26	Derivation of themes	The themes were derived from the data.	8
27	Software	Nvivo software were used for the coding.	8
28	Participant checking	The participants did not provide feedback on the findings.	NA
Reporting			
29	Quotations presented	Quotations presented with each paragraph, trying to illustrate the main points.	10-14
30	Data and findings consistent	The data and findings were cross-checked multiple times, ensuring consistency.	16
31	Clarity of major themes	Outlined in result table and in clear headings in the result section.	9-14
32	Clarity of minor themes	Outlined in result table and in clear headings in the result section.	9-14

Helldén D, *et al. BMJ Open* 2023; 13:e073853. doi: 10.1136/bmjopen-2023-073853

Full coding tables

Main themes and findings - full coding tables

**Theme**	SDGs and expanded view on child health enable change			
**Sub-themes**	Possibility for action due to SDGs			
**Categories**	Government commitment to and leadership of SDGs	SDGs provide a common vision and guide		Discrepancy between ambition and actual work
**Subcategories**		More detailed than MDGs	Showcase that health is a multisectoral issue	
**Codes**	Adoption and change of national plans and policies<br><br>No change in government as leaders of the goals<br><br>SDG implementation depends on alignment to government	Provide a clear set of goals<br><br>Provide a roadmap or guide<br><br>More detailed<br><br>More complex reflecting actual conditions	Illustrate that health is a multisectoral issue<br><br>SDGs reflecting actual conditions with regards to health	SDGs too complex, impossible to succeed<br><br>High ambition not matched with resources/work committed

Main themes and findings - full coding tables, continued.

**Themes**	SDGs and expanded view on child health enable change					
**Sub-themes**	Higher ambitions for child health, a multisectoral area at heart					
**Categories**	Definition of child health		Child health linkages across sectors			
**Sub-categories**	Child age under 18 years	A focus on not only health but well-being	Child health by definition a multisectoral issue	Education and schooling	Nutrition	General societal conditions
**Codes**	General view and legally a child is a person under 18 years of age	Physical and mental health equally important<br><br>Good nutrition and absence of disease	All SDGs important for child health<br><br>The linkages between sectors and child health cannot be divided	Education as most formative experience<br><br>School important physical place for linkages<br><br>Early child development key	Nutrition and functioning agricultural sector as basis for child growth<br><br>Commercial interests conflicts with good child nutrition	Physical safety and hygiene environment<br><br>Economic development of country<br><br>Social protection systems

Main themes and findings - full coding tables, continued.

**Theme**	SDGs and expanded view on child health enable change				
**Sub-themes**	Higher ambitions for child health, a multisectoral area at heart				
**Categories**	Aspects of the health system and actors unique to children			Special considerations for children	
**Sub-categories**	Responsibility of family and community	Influence of other actors	Key aspects of health system for improving child health	Life course approach	Enabling the child to thrive
**Codes**	Parents and family are the primary caretaker<br><br>Information and health literacy key undertaking<br><br>Social determinants of family dictates child health to large extent	Government overarching leader and supporter of child health<br><br>International organizations influence organizations in country<br><br>Commercial interests of private sector	Lack of focus on preventive child health measures<br><br>Need to improve quality and equity<br><br>Difference between rural and urban areas	Prenatal services important for child health<br><br>Children have different needs at different ages	A focus on child growth<br><br>Holistic approach<br><br>Acknowledging child rights

Helldén D, *et al. BMJ Open* 2023; 13:e073853. doi: 10.1136/bmjopen-2023-073853

Main themes and findings - full coding tables, continued.

**Theme**	Gap between theory and real world complexities					
**Sub-themes**	Linear process of collaboration					
**Categories**	Actors and topics			Identifying and framing problem		
**Sub-categories**	Broad variety of actors	Territory feelings	Collaborations focused on non-health aspects	Top-down approach	Bottom-up approach	Framing of problem
**Codes**	Government as natural leader<br><br>Civil society networks<br><br>External donors emphasize importance<br><br>Many different actors collaborating	There exist strict boundaries between actors<br><br>Competition between actors for funding<br><br>Skeptical view of government and NGO and vice versa	Focused on preventive issues<br><br>Collaboration indirectly see effect on child health<br><br>Willingness to connect to child health	Government or ministries identifies need<br><br>National policy or development plan<br><br>International agenda or external funding opportunities<br><br>From own organizational strategy or values	Listening to stakeholders in community or on sub-national level<br><br>Routine data or findings from actual situation on the ground<br><br>Reliable data not always present	Research as a way of narrowing problem<br><br>Involving many actors in collective process<br><br>Detailed problem statement

Main themes and findings - full coding tables, continued.

**Theme**	Gap between theory and real world complexities						
**Sub-themes**	Linear process of collaboration						
**Categories**	Planning			Coordination			
**Sub-categories**	Complex, detailed, resource constraining process	Capacity assessment key	Prioritization depending on context	Varied methods of coordination	Clear division of responsibilities	Leadership paramount	Power and hierarchies influence coordination
**Codes**	Many actors involved in planning<br><br>Sub-national and national level engaged<br><br>Technical level and strategy level<br><br>Detailed collaboration plan and outline of activities, outputs,	Technical skill and resource capacity at implementer level instrumental<br><br>Division of activities based on capacity<br><br>If not enough capacity collaboration cannot begin	Prioritization based on funding requirements<br><br>Politics and benefits of including certain actors or activities	Information sharing mechanisms<br><br>Focal points or joint committees<br><br>Regular, continuous coordination<br><br>Built on existing structures	Agreed upon plan of responsibilities<br><br>Common vision and commitment key for ease of coordination<br><br>Participation in joint coordination hard	Single organization that explicitly or implicitly lead<br><br>Structuring collaboration efforts depends on leader	Focal points for collaboration lack decision making power<br><br>Power imbalance due to government more powerful<br><br>Competing for funding between organizations

Helldén D, *et al. BMJ Open* 2023; 13:e073853. doi: 10.1136/bmjopen-2023-073853

	and desired outcomes<br><br>Commitment and ownership implicit goals of process						

Helldén D, *et al. BMJ Open* 2023; 13:e073853. doi: 10.1136/bmjopen-2023-073853

Main themes and findings - full coding tables, continued.

Theme	Gap between theory and real world complexities						
**Sub-themes**	Linear process of collaboration						
**Categories**	Implementation			Monitoring and evaluation			
**Sub-categories**	Adaptability to change	Geographical and administrative level	Follows from planning and coordination	Detailed but depends on funding	Hard to move beyond outputs	Integral to the collaboration	Responsibility for M&E varies
**Codes**	Implementation does not follow plan<br><br>Funding changes requires change of plan<br><br>Government involvement lead to less flexibility<br><br>Covid-19 disruption	Focus on implementing organizations or participations<br><br>Added complexity for actual implementation<br><br>National level collaboration, sub-national implement<br><br>Sub-national own system of priorities,	Implementation mirrors previous collaborative efforts<br><br>Reduction in parallel work and efficient implementation<br><br>Takes time and resources to implement, need to be considered before start	Funding source and resources key for allowing M&E<br><br>M&E include detailed indicators<br><br>Government or external donor relies heavily on M&E for decisions	Discrepancy in M&E between stakeholders<br><br>Particularly hard to attribute success or failures<br><br>Quantitative indicators more favorable	Learning from failure<br><br>M&E seen as opportunity to learn and improve<br><br>Successes can build momentum, secure resources<br><br>Serves as main accountability mechanism	Internal or external evaluation depending on context and resources<br><br>One stakeholder monitors activities<br><br>Joint monitoring of activities

		relationships and focus					

Main themes and findings - full coding tables, continued.

Theme	Gap between theory and real world complexities						
Sub-themes	Linear process of collaboration		Real-world complexities shaping the collaboration				
Categories	Dissemination		Funding		Relationships		
Sub-categories	Information spreading	Recognition	Call for more funding	Funding as a source of power	Facilitate or hamper collaboration	Actively building relationships	Relationships as an outcome
Codes	Engage the public and stakeholders<br><br>Increase awareness	Engaging national-level government<br><br>Gain international reputation	Budget greatest limitation to collaboration<br><br>Not enough government/national funding<br><br>Funding sources varies<br><br>If government funding more sustainable	External donors agenda decide activities<br><br>If funding from government they have last say<br><br>Leadership often based on funding<br><br>Ministry of economy key stakeholder	Tensions between NGOs and government evident<br><br>Conflicts within government or NGO networks<br><br>Common understanding and relationships increase coordination	Continuous relationship building<br><br>Efforts by stakeholders to build relationships	Over time relationships built through coordination meetings and implementation<br><br>Evolve between key focal points<br><br>Mutual understanding lead to trust and confidence

BMJ Publishing Group Limited (BMJ) disclaims all liability and responsibility arising from any reliance placed on this supplemental material which has been supplied by the author(s)

				Decide design and coordination of collaboration			

Main themes and findings - full coding tables, continued.

**Theme**	Gap between theory and real world complexities									
**Sub-themes**	Real-world complexities shaping the collaboration					Critically assessing collaborations				
**Categories**	Enabling environment		Capacity building			Success factors			Obstacles	
**Sub-categories**	Policies	Government	Actual method depends on collaboration	Key for sustainability	Demands resources	Clear responsibilities	Common vision and understanding	Secure buy in	Real world complexities	Lack of accountability
**Codes**	International agenda facilitate work<br><br>Sub-national plans for development<br><br>National CSDG roadmap and other national plans	Active and collaborative government ministries<br><br>Existing multisectoral ministerial committees	In person technical capacity building<br><br>Natural reciprocal<br><br>Effort to include capacity building<br><br>Capacity building according to administrative	Learning and incorporating changes<br><br>Integral part of collaboration itself, one of main benefits<br><br>Building capacity with implementors or sub/national level lead to sustainability	Capacity building takes time<br><br>Capacity building limited by funding	Agreement on division of activities<br><br>Leadership from all<br><br>Functioning M&E	Learning continuously<br><br>Open sharing and discussion<br><br>Benefits and goals explicit	Engage stakeholders from beginning<br><br>Government and sustainable funding<br><br>Commitment from all<br><br>Relationship and capacity building	Lack of funding, sustainability<br><br>Politics on sub-national and national level<br><br>Competing priorities and work	No commitment to work together<br><br>Lack of transparency<br><br>Difficulty of attributing failures or successes

			and geographical level							

Examples of multisectoral collaboration that include child health and well-being in Cambodia

Name	Short description	Source
Multisectoral Food and Nutrition Security in Cambodia (MUSEFO)	Through a multisectoral approach, the programme aims to improve the nutrition of women and young children through<br>i) Improving the quality of nutrition services by providing training for health workers.<br>ii) Diversifying nutrition and food production by providing trainings for farmers, building their capacity to grow a more diverse range of crops and improving their access to healthy foods.<br>iii) Embedding successful approaches on national and regional level is the third field of action.	https://giz-cambodia.com/wordpress/wp-content/uploads/10_FactSheet-of-Multisectoral-Food-and-Nutrition-Security-in-Cambodia-MUSEFO.pdf
Identification of Poor Households Programme (ID Poor)	The ID Poor program aim to identify at risk or poor households in Cambodia and provide Equity Cards to these households as a basis for assessing social assistance services. This can then be used by various ministries or other organizations to assist at-risk households with healthcare for children.	https://idpoor.gov.kh/en/
The Second National Strategy for Food Security and Nutrition 2019-2023	Acknowledging the cross-cutting challenges facing the ambition to provide proper food and nutrition, including promoting infant breastfeeding practices, the government has implemented a national-wide strategy which explicitly take an multisectoral approach to nutrition.	https://scalingupnutrition.org/sites/default/files/2022-06/national-nutrition-plan-cambodia.pdf
Raising Awareness and Innovative	Led by Save the Children, the project used innovative approaches to increase awareness and appreciation of a	https://resourcecentre.savethechildren.net/pdf/RAISE-Evaluation-Report.-Final.-16-March-2022-1.pdf/

Strategies for ECD (RAISE)	holistic approach to early childhood development in 43 villages in Kampong Siem district.	
Family Care First (FCF) and Responsive and Effective Child Welfare Systems Transformation (REACT)	Facilitated by Save the Children, the project is a multi-donor supported network of organizations including government, NGO´s and UN organizations working together to support children to live in safe, nurturing family-based care. The work take place across numerous sectors and stakeholders.	https://resourcecentre.savethechildren.net/pdf/ Gender%20Intersectionality%2 0and%20 Family%20Separation%20Alternative%20Care%20and%20%20the%20 Reintegration%20of20Children%20FINAL_0.pdf/
The Fifth National Strategic Plan for a Comprehensive, Multi-Sectoral Response to HIV/AIDS (2019-2023)	Through a multisectoral approach and partnership across ministries and different organizations, Cambodia is working towards the 90-90-90 targets and eventually elimination of new HIV infections including mother-to-child transmission.	http://www.healthpolicyplus.com/ns/pubs/17402-17725_CambodiaStrategicPlan.pdf

Helldén D, et al. BMJ Open 2023; 13:e073853. doi: 10.1136/bmjopen-2023-073853
