# Supplementary material for: Sustainable development goals and multisectoral collaborations for child health in Cambodia: a qualitative interview study with key child health stakeholders
Source: BMJ Open. 2023 Nov 21;13(11):e073853. doi: 10.1136/bmjopen-2023-073853 (PMC10668300; doi:10.1136/bmjopen-2023-073853)
Supplement: Supplementary data [file bmjopen-2023-073853supp002.pdf]

Supplementary Material 2. Reflexivity Statement

Study conceptualisation:

1. How does this study address local research and policy priorities?

This study is a part of an effort to provide country specific knowledge of multisectoral collaborations in Cambodia, a key knowledge gap identified by stakeholders and academia. The government and other organizations are actively engaging in multisectoral collaborations, and understanding how the function in practice is a key priority.

2. How were local researchers involved in study design?

The local researchers (SS and TC) were engaged in the overall design of the study and particularly the identification and recruitment of participants as well as development of the interview guides and data collection. They were core members of the study team.

Research management:

1. How has funding been used to support the local research team(s)?

The study was funded through the Swedish Research Council (2018-03609) with the majority of funding dedicated to country study activities and local research colleagues (SS and TC).

Data acquisition and analysis:

1. How are research staff who conducted data collection acknowledged?

The researchers who conducted data collection met the authorship criteria and are hence acknowledged as co-authors of the study.

2. How have members of the research partnership been provided with access to study data?

All members of the research team, including SS and TC, had full access to the data.

3. How were data used to develop analytical skills within the partnership?

The qualitative data analysis was conducted by DH with input and training of DH, SS and TC by a qualitative research expert (HMA).

Data interpretation:

1. How have research partners collaborated in interpreting study data?

The results from the study were continuously discussed with the local research colleagues (SS and TC) who contributed significantly to the interpretation of the results.

Drafting and revising for intellectual content:

1. How were research partners supported to develop writing skills?

Most of the writing of the manuscript was done by DH, however local research colleagues (SS and TC) provided crucial input.

2. How will research products be shared to address local needs?
The results from the study will be disseminated widely to an international and national audience, including a dissemination seminar with relevant country stakeholders.

Authorship:

1. How is the leadership, contribution and ownership of this work by LMIC researchers recognized within the authorship?
The local researchers (SS and TC) authors 2-3, recognizing their crucial hands-on contribution to the study.

2. How have early career researchers across the partnership been included within the authorship team?
The first author is a PhD student (although not from a LMIC), SS and TC are recognized experienced researchers.

3. How has gender balance been addressed within the authorship?
Out of the seven authors, four are male (DH, SS, TC and TA) while three (HN, SK and HMA) are female. The preponderance for male authors is weighted against the critical study design and interpretation by HN and SK while HMA is a world-leading qualitative expert.

Training:

1. How has the project contributed to training of LMIC researchers?
The LMIC researchers (SS and TC) are experienced qualitative researchers, however within this study all authors gained refresher trainings and developed their qualitative analytical skills and knowledge of framework method analysis by HMA (qualitative expert).

Infrastructure:

1. How has the project contributed to improvements in local infrastructure?
No direct benefit in local infrastructure has come from this qualitative study, however the findings of the study can help to conceptualize and form partnerships across sectors that can lead to improvements in infrastructure.

Governance:

1. What safeguarding procedures were used to protect local study participants and researchers?
The study conforms to the Helsinki declaration and followed the ethical and practical guidelines stipulated by the National Ethics Committee for Health Research in Cambodia regarding the safety of researchers and participants.
