# Supplementary material for: Sustainable development goals and multisectoral collaborations for child health in Cambodia: a qualitative interview study with key child health stakeholders
Source: BMJ Open. 2023 Nov 21;13(11):e073853. doi: 10.1136/bmjopen-2023-073853 (PMC10668300; doi:10.1136/bmjopen-2023-073853)
Supplement: Author's manuscript [file bmjopen-2023-073853.draft_revisions.pdf]

BMJ Open is committed to open peer review. As part of this commitment we make the peer review history of every article we publish publicly available.

When an article is published we post the peer reviewers' comments and the authors' responses online. We also post the versions of the paper that were used during peer review. These are the versions that the peer review comments apply to.

The versions of the paper that follow are the versions that were submitted during the peer review process. They are not the versions of record or the final published versions. They should not be cited or distributed as the published version of this manuscript.

BMJ Open is an open access journal and the full, final, typeset and author-corrected version of record of the manuscript is available on our site with no access controls, subscription charges or pay-per-view fees (http://bmjopen.bmj.com).

If you have any questions on BMJ Open's open peer review process please email info.bmjopen@bmj.com

BMJ Open

Sustainable development goals and multisectoral collaborations for child health in Cambodia: a qualitative interview study with key child health stakeholders

Journal:	*BMJ Open*
Manuscript ID	bmjopen-2023-073853
Article Type:	Original research
Date Submitted by the Author:	20-Mar-2023
Complete List of Authors:	Helldén, Daniel ; Karolinska Institutet, Department of Global Public Health<br>Sok, Serey; Royal University of Phnom Penh, Research Office<br>Chea, Thy; Malaria Consortium<br>Nordenstedt , Helena; Karolinska Institute, Department of Global Public Health<br>Kuruvilla, Shyama ; WHO International<br>Alvesson, Helle; Karolinska Institutet, Department of Global Public Health<br>Alfvén, Tobias ; Karolinska Institute, Global Public Health; Sachs´ Children and Youth Hospital
Keywords:	Health policy < HEALTH SERVICES ADMINISTRATION & MANAGEMENT, Community child health < PAEDIATRICS, Organisation of health services < HEALTH SERVICES ADMINISTRATION & MANAGEMENT

SCHOLARONE™
Manuscripts

I, the Submitting Author has the right to grant and does grant on behalf of all authors of the Work (as defined in the below author licence), an exclusive licence and/or a non-exclusive licence for contributions from authors who are: i) UK Crown employees; ii) where BMJ has agreed a CC-BY licence shall apply, and/or iii) in accordance with the terms applicable for US Federal Government officers or employees acting as part of their official duties; on a worldwide, perpetual, irrevocable, royalty-free basis to BMJ Publishing Group Ltd ("BMJ") its licensees and where the relevant Journal is co-owned by BMJ to the co-owners of the Journal, to publish the Work in this journal and any other BMJ products and to exploit all rights, as set out in our licence.

The Submitting Author accepts and understands that any supply made under these terms is made by BMJ to the Submitting Author unless you are acting as an employee on behalf of your employer or a postgraduate student of an affiliated institution which is paying any applicable article publishing charge ("APC") for Open Access articles. Where the Submitting Author wishes to make the Work available on an Open Access basis (and intends to pay the relevant APC), the terms of reuse of such Open Access shall be governed by a Creative Commons licence – details of these licences and which Creative Commons licence will apply to this Work are set out in our licence referred to above.

Other than as permitted in any relevant BMJ Author's Self Archiving Policies, I confirm this Work has not been accepted for publication elsewhere, is not being considered for publication elsewhere and does not duplicate material already published. I confirm all authors consent to publication of this Work and authorise the granting of this licence.

For peer review only

Title: Sustainable development goals and multisectoral collaborations for child health in Cambodia: a qualitative interview study with key child health stakeholders

Authors: Daniel Helldén[1]*, Serey Sok[2], Thy Chea[3], Helena Nordenstedt[1], Shyama Kuruvilla[4], Helle Mölsted Alvesson[1], Tobias Alfvén[1,5]

Affiliations:

[1]Department of Global Public Health, Karolinska Institutet, Stockholm, Sweden

[2]Research Office, Royal University of Phnom Penh, Phnom Penh, Cambodia

[3]Malaria Consortium, Phnom Penh, Cambodia

[4]World Health Organization, Geneva, Switzerland

[5]Sachs' Children and Youth Hospital, Stockholm, Sweden

Keywords: multisectoral collaboration, health policy, child health, SDGs

Number of

Tables: 2

Figures: 0

Words: 4608

[1] Affiliation: Department of Global Public Health, Karolinska Institutet, Stockholm, Sweden
Address: Tomtebodavägen 18 A, SE-171 77 Stockholm, Sweden
Email: daniel.hellden@ki.se
Telephone: +46732406555

For peer review only - http://bmjopen.bmj.com/site/about/guidelines.xhtml

Abstract

Objectives: Multisectoral collaboration highlighted as key in delivering on the Sustainable Development Goals (SDGs), but still little is known on how to move from rhetoric to action. Cambodia has made remarkable progress on child health over the last decades with multisectoral collaborations being a key success factor. However, it is not known how country stakeholders perceive the SDGs, the concept of child health in the context of the SDGs and multisectoral collaborations for child health in Cambodia.

Methods: Through purposive sampling, we conducted semi-structured interviews with 29 key child health stakeholders from a range of government and non-governmental organisations. Guided by framework analysis, themes, sub-themes and categories were derived.

Results: We found that the adoption of the SDGs led to increased possibility for action and higher ambitions for child health in Cambodia, while simultaneously establishing child health as a multisectoral issue. There seem to be a discrepancy between the desired step-by-step theory of conducting multisectoral collaboration and the real-world complexities including funding and power dynamics that heavily influence the process of collaboration. Identified success factors for multisectoral collaborations included having clear responsibilities, leadership from all and trust among stakeholders while the major obstacle found was lack of sustainable funding.

Conclusion: The findings from this in-depth multistakeholder study can inform policy makers and practitioners on the theoretical and practical process as well as influencing aspects that shape multisectoral collaborations in general and for child health specifically. This is vital if multisectoral collaborations are to be successfully leveraged to accelerate the work towards achieving better child health in the era of the SDGs.

Strengths and limitations

- This is the first study to provide in-country insights on multisectoral collaborations for child health that can be transferable to similar settings.

- We have explored the themes surrounding multisectoral collaboration for child health broadly, capturing important discrepancies, success factors and obstacles through the semi-structured interviews with a relatively large sample of child health stakeholders.

- The sample participants interviewed is unbalanced in terms of gender and expertise in different SDG areas.

For peer review only

For peer review only - http://bmjopen.bmj.com/site/about/guidelines.xhtml

INTRODUCTION

Almost halfway until the United Nations Sustainable Development Goals (SDGs) are to be achieved, practitioners, experts and policy makers are trying to speed up the pace of progress on child health. This has become even more urgent with the setback of the COVID-19 pandemic which left 147 million children out of proper education, rising child labor and significantly higher rates of malnutrition and over 22 million children missing essential vaccinations.[1,2] Over the last decades it has become evident that progress made in other sectors heavily impact the possibility to make progress on child health and well-being.[3,4] Further, progress on child health and well-being are essential for tackling poverty and promote the development of societies.[5] Moving beyond mere child survival, there is now a larger focus on enabling children to thrive and reach their full potential.[5,6]

Multisectoral collaborations have long been seen as critical for achieving gains in health and well-being when it comes to universal health coverage, non-communicable diseases and succeeding in governing multisectoral issues going back to the World Health Organization (WHO) Constitution and the Alma Ata Declaration.[7–9] For child health a multisectoral approach to areas such as nutrition[10] and education[11,12] have been studied, however there is lack of understanding how multisectoral collaborations work out on a country level. Further, a generic analysis of the linkages between the SDGs and child health found that there are many synergies between making progress on the SDGs and accelerating progress on child health, suggesting that multisectoral collaboration could harness synergies and better handle tradeoffs between the SDGs and child health.[13]

During the Millennium Development Goal era many countries made significant gains in child health, and approximately half of the reduction in child mortality between 1990 and 2010 have been attributed to investments in sectors outside of health.[14] Cambodia was one of the fast-track countries and made significant progress including succeeded in lowering the under-five mortality from 116 to 29 deaths per 1000 live births from 1990 to 2015.[15] Many challenges persist however, with significant inequalities between rural and urban areas, lower than desired educational attainment and sub-optimal water and sanitation conditions in schools and residential areas.[16] It has been shown that multisectoral efforts, such as the ID Poor program, have been successful in reducing poverty and collaborative initiatives between non-health sectors have become a cornerstone of the maternal and child health strategy in Cambodia.[17–19]

Cambodia has managed to improve the health and well-being of children over a short period of time while utilizing collaborations across sectors to do so among other changes. However, it is not known how child health stakeholders have been influenced by the SDGs or how they theorize multisectoral collaborations, here defined as “multiple sectors and stakeholder intentionally coming together and collaborating in a managed process to achieve shared outcomes and common goals”[20], versus the actual practice of conducting such collaborations. Hence, our aim was to understand how stakeholders in Cambodia perceive the SDGs, child health in the era of the SDGs and multisectoral collaborations for child health in Cambodia.

For peer review only - http://bmjopen.bmj.com/site/about/guidelines.xhtml

METHODS

Study design and setting

Guided by the The COnsolidated criteria for REporting Qualitative (COREQ) recommendations[21] and the concept of information power[22] this study utilizes semi-structured interviews to investigate how Cambodian stakeholders perceive the SDGs, the concept of child health in the era of the SDGs and multisectoral collaborations for child health in Cambodia. The country is governed primarily through the national government, which consist of the council of ministers led by the prime minister while the parliament (national assembly and senate) have legislative power. Administratively the country is divided into provinces, districts, communes and villages.[23] During the last decade, the government has incrementally favored a more decentralized approach where districts and commune government officials are given more funding and implementing power.[24] Collaboration between government and non-government stakeholders on primarily occur on two levels, the national or sub-national (district or commune) level and been characterized by an increased role of the government in leading and coordinating collaborations.[25,26]

Participant identification and recruitment

Key child health stakeholders with country specific knowledge as well as non-health sector stakeholders on a national level in Cambodia were identified for participation by the research team. Participants were purposively selected based on predefined criteria of having expertise in child health or being from a non-health sector (for example water and sanitation, agriculture, infrastructure etc.) but with implementation knowledge of how child health interacts with other sectors in Cambodia. Efforts were made to recruit participants from many different sectors, including having participants from inside and outside of government. Further, the recruitment of participants was aimed to be balanced in terms of sex and seniority. The expected total number of participants was 30, balancing the need for reaching satisfactory information power[22] and feasibility.

Data collection

A total of 29 participants were interviewed between April-June 2020. The characteristics of the participants can be found in **Table 1**. Information was given verbally to all participants on the purpose of the study, what their involvement in the study would be, the risks and benefits of taking part in the study, and that they had the right to decline participation or withdraw from the study at any time for any reason. Participants were asked to sign an informed consent form, written in Khmer before the interview started. The interviews were held in Khmer by authors SS and TC, audio recorded and transcribed verbatim into English. The interviews took place in Phnom Penh city vicinity, at the participant´s place of employment or other convenient but private location for the participant. An interview guide was developed based on established multisectoral frameworks; the SDG Synergies framework[27], Health in all policies approach[28] and multisectoral collaborative model presented by Kuruvilla et al[20] (see **Supplementary Material 1** for interview guide). The interview started with general background information on the participant, the perception of the SDGs, child health and multisectoral collaboration and then focused on multisectoral collaboration for child health within the Cambodia context (identification of problem, design, implementation, and monitoring of the

For peer review only - http://bmjopen.bmj.com/site/about/guidelines.xhtml

146 collaboration as well as relationships and capacity building activities). Two pilot interviews were held
147 where after the interview guide was slightly adjusted for clarity.

For peer review only

For peer review only - http://bmjopen.bmj.com/site/about/guidelines.xhtml

Table 1. Participant characteristics

Nr	Sex	Years worked	Organisation	Experience according to Cambodian Sustainable Development Goals																	
				1	2	3	4	5	6	7	8	9	10	11	12	13	14	15	16	17	18
1	Male	6-14	Governmental																		
2	Female	>15	Governmental																		
3	Female	>15	Governmental																		
4	Male	>15	Governmental																		
5	Male	>15	Governmental																		
6	Female	1-5	Governmental																		
7	Male	>15	Governmental																		
8	Male	>15	Governmental																		
9	Male	>15	Governmental																		
10	Male	>15	Governmental																		
11	Male	>15	Governmental																		
12	Male	6-14	Governmental																		
13	Female	6-14	Governmental																		
14	Male	6-14	Governmental																		
15	Male	6-14	Non-governmental																		

For peer review only - http://bmjopen.bmj.com/site/about/guidelines.xhtml

16	Male	>15	Non-governmental																		
17	Male	>15	Non-governmental																		
18	Male	6-14	Non-governmental																		
19	Female	1-5	Non-governmental																		
20	Male	>15	Non-governmental																		
21	Male	>15	Non-governmental																		
22	Female	>15	Non-governmental																		
23	Male	>15	Non-governmental																		
24	Female	>15	Non-governmental																		
25	Male	>15	Non-governmental																		
26	Male	>15	Non-governmental																		
27	Male	6-14	International																		
28	Female	6-14	International																		
29	Male	>15	International																		

For peer review only

For peer review only - http://bmjopen.bmj.com/site/about/guidelines.xhtml

Data analysis

Transcripts were imported into NVivo software for analysis. The transcripts were first analyzed by framework method analysis[29] by which the transcripts were read in full by DH, then coded through identification of meaning units, combining these into sub-categories and then grouped into overarching categories and lastly themes following the standard methodology. The themes, categories and sub-categories were inductively developed without prior anticipations [30] and continuously developed during the review of the transcripts. The coding was cross-checked by HMA and the analysis was continuously discussed with SS and TC to improve trustworthiness and validity.[22]

Patient and public involvement

No patients or public representative were directly involved in the design, conduct or reporting of this study. The findings will be disseminated and discussed with involved stakeholders. A reflexivity statement can be found in the **Supplementary Material 2.**

RESULTS

A diverse set of perspectives on the research questions out of which two main themes were developed in addition to several sub-themes and categories (**Table 2**, see **Supplementary Material 1** for full coding tables and COREQ checklist). The first theme related to the views of the participants on how the SDGs and expanded view on child health enable change the and the second main theme detailed the gap between theory and real-world complexities of conducting multisectoral collaborations for child health.

For peer review only

Table 2. Main themes, sub-themes and categories

**Themes**	SDGs and expanded view on child health enable change		Gap between theory and real-world complexities		
**Sub-themes**	Possibility for action due to SDGs	Higher ambitions for child health, a multisectoral area at heart	Planned linear process of collaboration	Real-world complexities shaping the collaboration	Critically assessing collaboration
**Categories**	SDGs provide a common vision and guide<br><br>Government commitment to and leadership of SDGs<br><br>Discrepancy between ambition and actual work	Definition of child health<br><br>Child health linkages across sectors<br><br>Aspects of the health system and actors unique to children<br><br>Special considerations for children	Identifying and framing problem<br><br>Actors and topics<br><br>Planning<br><br>Coordination<br><br>Implementation<br><br>Monitoring and evaluation<br><br>Dissemination	Funding<br><br>Relationships<br><br>Enabling environment<br><br>Capacity building	Success factors<br><br>Obstacles

For peer review only - http://bmjopen.bmj.com/site/about/guidelines.xhtml

For peer review only

SDGs and expanded view on child health enable change

Overall, interviewees reflected on the willingness by the government to adopt the SDGs, how the possibility to achieving the SDGs depends on the outlook for the country while concluding that child health is a multisectoral topic at heart and that with the introduction of the SDGs the participants had set higher ambitions for child health and well-being.

Possibility for action due to SDGs

The 2030 Agenda and the SDGs were thought of as a universally relevant vision for sustainable development, providing a concrete roadmap or guide for each country. Comparing with the previous Millennium Development Goals, participants reflected on how the SDGs represent a more complex and detailed set of objectives that mirror actual conditions in the country. There was an overall agreement that the SDGs showcase that health is a multisectoral issue more clearly than during the Millennium Development Goals era. However, although the commitment to and leadership of the government of Cambodia in adopting and implementing the Cambodian Sustainable Development Goals were evident, some participants noted the discrepancy between the highly set ambitions of the contextualised SDGs with the resources and work committed.

"That's the difference in perspectives between policymakers and implementers. The implementers in the ministry will complain about having lots of challenges and risks which could lead to a lower result. So, the plan to achieve many things by 2030 has already been written down. However, the implementation need budget and solutions to the challenge." - Nr 21, non-governmental organisation

Higher ambitions for child health, a multisectoral area at heart

Focusing on child health, most regarded children as people under the age of 18 and emphasised that physical and mental health are of equal importance to children. Interviewees detailed a range of linkages between child health and other sectors, mostly focusing on education and schooling, nutrition and other general societal conditions such as physical safety, environment, economic development and social protection systems. Overall, there was a strong notion of indivisibility between child health and its determinants, making the case that child health by definition is a multisectoral issue with all sectors responsible for its improvement.

"Like I mentioned, child health consists of physical, mental and social health. So, we need all relevant institutions to improve physical, mental and social health. We can't miss anyone to work on it." - Nr 5, governmental organisation

Interviewees put an emphasis on the family as responsible for the child´s health, while other stakeholders (government, international organisations and private sector) play an important role in shaping the determinants of child health in Cambodia. They further urged a concrete focus on preventive measures, improving quality and reach of health services related to the child and the family to improve child health further. Lastly, interviewees made the case for a life course approach to child health and setting a higher ambition for children with a focus on child growth and stronger acknowledgment of the rights of the child.

"To understand about the needs of children, we need to understand the growth of them first. Children's development consists of children before birth, children after birth to two years old, children in

For peer review only - http://bmjopen.bmj.com/site/about/guidelines.xhtml

kindergarten and primary school, and children in high school. The development of children on physical health, education and morality are ongoing process." - Nr 5, governmental organisation

Gap between theory and real-world complexities

When discussing multisectoral collaborations for child health, it became clear that there is a step-by-step linear process of thinking around the collaboration and its activities while aspects influencing the collaboration shape and direct the process in non-linear fashions. Participants also critically assess the collaborations, identifying success factors and obstacles for these types of collaborations in Cambodia.

Planned linear process of collaboration

The beginning of a multisectoral collaboration typically began with the identification and framing of a problem. This could be from a top-down approach, whereby government ministries identified a gap or need, or through policy or development plans while funding opportunities and the own organisational strategy or values could be other ways of identifying a problem. On the other hand, interviewees also described a bottom-up approach of problems being identified through routine data or findings from the grassroot level, complemented by listening and learning from community or sub-national stakeholders. The identified problem was often not primarily concerned with health but noted that child health might stand to benefit as an effect of a successful solution to the problem. The problem was typically framed in a detailed problem statement following involvement of many stakeholders in collective process, often using research in some way to narrow the problem.

"So, the needs can be identified through annual reports and through our observation in different sectors. Sometimes, we also do things following the donors' research and findings." – Nr 1, governmental organisation.

"They (government officials) collected all data from institutions under Ministry of Health. Then, they identified the challenge and priority action plans for next year. Besides, each unit need to monitor their annual results and to identify the priority action plans. That's how the Ministry of Health and different units identify the needs on child health, status, results and ways forward to reach SDGs."- Nr 6, governmental organisation.

The stakeholders involved in the discussed collaborations varied substantially, however the government was seen as a natural leader of collaborations while non-governmental organisations often organised in networks. Interviewees expressed territory feelings, with relatively strict boundaries between stakeholders and a critical view of government by the non-governmental organisations and vice versa.

"I am not blaming the government institutions, but there are some institutions which have too clear boundaries on their responsibilities and work. This leads to failure in our work. " – Nr 29, international organisation.

Planning of the collaboration were seen as a complex, detailed and resource demanding process. Often not formalised, a capacity assessment of the stakeholders in the collaboration, primarily focusing on

For peer review only - http://bmjopen.bmj.com/site/about/guidelines.xhtml

implementation capacity, were seen as key with the division of activities based on this assessment. If there was not enough capacity to solve the problem identified, the collaboration could not begin. During the planning process interviewees noted that prioritization of activities was done depending on the funding requirements and to secure buy-in from certain stakeholders (particularly government) seen as necessary for the success of the collaboration.

"For example, they (government servants) may plan 20 activities, but receive inadequate budget. So, they prioritize the activities to be done. According to my observation, district level is the same. They engage politics into their work. They like infrastructure development more than social development because it is eye-catching and visible." – Nr 9, governmental organisation.

Coordination was done in various ways depending on the collaboration however there were usually a common information sharing mechanisms, focal points at each stakeholder or joint committees with continuous coordination often built on somewhat already existing structures. There was also a clear division of responsibilities, although participation in joint coordination could be difficult to achieve and often those who coordinate do not have decision making power. Clear leadership of the collaboration was seen as paramount, with coordination succeeding or faltering based on the competence and willingness of the leader. As such, coordination was both a formal and informal process. Indeed, power and hierarchies shaped the coordination efforts where power imbalances or competition for funds between stakeholders could threaten the whole collaboration.

"Those people also need to have the authorization in decision-making in the meeting. In the past, there were people who attended the meeting, but did not do what were discussed. It was useless when people came to listen, but didn't share to their management and colleagues." – Nr 15, non-governmental organisation.

Implementation of the collaboration tried to follow the planning and set coordination mechanisms. However, collaborations were able to change depending on a change in the context or influencing aspects such as the Covid-19 pandemic or funding changes. Interviewees emphasised the difference between the national and sub-national level in terms of the collaboration, with larger collaborations having an administrative or policy function at the national level while implementation occurred at the sub-national level. This structure often led to increased complexities, with a different set of stakeholders needing to be involved at the different levels and the sub-national system having its own set of priorities.

"National level only work on policy. So, implementation goes to community level. I think that we should focus on provincial and communal level first to let them implement the work. We should also try to integrate the coordination with national level too by using forum to meet and discuss on the challenge." - Nr 28, international organisation.

Monitoring and evaluation were seen as integral to the collaboration, enabling learning and improvement of the collaboration itself and its activities and serving as the main accountability mechanism. The responsibility of conducting the monitoring and evaluation varied depending on the context and funding available, with external evaluation being seen as favourable if it could be funded. The government and international organisations relied heavily on monitoring and evaluation for making decisions about the collaborations. However, it was seen as hard to move beyond pure outputs, with quantitative indicators believed to be most reliable, and to attribute successes or failures to different stakeholders in the collaboration.

For peer review only - http://bmjopen.bmj.com/site/about/guidelines.xhtml

Dissemination of the collaboration and its activities were primarily thought of as information spreading, trying to raise awareness of the identified problem and engage the public and relevant stakeholders at national and international level in the efforts to solve it. It was also deemed important as a means of ensuring recognition from national level government ministries or the international community for the work done.

"We shared a lot, especially early childhood development program. We shared at provincial level and national committee on children education. We invited those committees to see our target location and our work. So, we disseminated a lot." Nr 22, non-governmental organisation.

Real-world complexities shaping the collaboration

There were a range of aspects influencing the process, often challenging the idea of a step-by-step linear approach of the collaboration. The most prominent aspect throughout was the funding, interviewees described the budget as the greatest limitation to the collaboration and called for more governmental funding at the national and sub-national level for multisectoral collaborations. Funding was seen as the most important source of power in the collaboration. Leadership roles, agenda setting and decision-making were mostly done by the organisation that controlled the funding.

"More importantly, we need the money to be available at sub-national level. The partners are all institutions. If the government can't manage to work on everything, we can ask civil society to help working on that. Nowadays, we are sceptical with NGOs. But, we also have example of government providing budget for NGOs to work." Nr 4, governmental organisation.

Relationships between the collaborators could facilitate or hamper the collaboration, with tensions between non-governmental organisations and the government existing and at the same time conflicts between government ministries or civil society networks that added complexity. For this reason, many collaborations tried to actively build relationships over time particularly between coordination focal points or joint committees, seeing mutual understanding leading to trust and confidence in the collaboration.

"The collaborative work also became better. During my time at education sectors, the relationship between partners was going very well, and we were happy to share any documents or data." – Nr 23, non-governmental organisation.

"There are many NGOs working to promote children. The government don't even know who they are. Some NGOs don't care about networking with the government too. This is the challenge according to my observation as a person in the middle of the two institutions. Both have their own weakness. Some NGOs do not know what the ministry have. For example, some NGOs do not know about existing guideline, plan or projects to work consistently. They only focus on their own work, and not pay attention to what others do to work collaboratively on the topic." – Nr 2, governmental organisation.

Capacity building were deemed to be key for the sustainability of the collaboration and its activities, particularly at the sub-national or implementation level, although demanding significant resources and the actual method varied depending on the type of collaborations and the stakeholders involved.

"Whenever there are requests from anyone or any organisations, we always respond and provide the training or sharing of experiences. We never hide our knowledge. We don't even charge them. We do it from our heart and soul. " – Nr 10, governmental organisation.

For peer review only - http://bmjopen.bmj.com/site/about/guidelines.xhtml

An enabling environment, particularly concerning policy and governmental direction within which the collaboration took place, were seen as being of crucial importance. The introduction and adoption of the 2030 Agenda and the Cambodia Sustainable Development Goals, sub-national plans for development and national level plans promoted the idea of multisectoral collaboration. Government ministries that actively promoted or worked in multisectoral ways or through multisectoral committees, albeit not always successful, further promoted the advantages of tackling problems in a multisectoral fashion.

"The main thing is whether or not they have the commitment to work together. When commitment on that occur, the work can be done easily because visions created in country and global level has already been created." – Nr 17, non-governmental organisation.

Critically assessing collaborations

Interviewees reflected critically on their collaborations and had through experience identified some key success factors and often faced obstacles of multisectoral collaborations in Cambodia. Having clear responsibilities with agreement on division of activities, leadership from all and functioning monitoring and evaluation as well as a common vision and understanding based on continuous learning in an open environment where benefits and goals were explicit seem to be key success factors. Further, many emphasised the necessity of securing buy in, trust and commitment from all stakeholders in the collaboration from the beginning with the government having a special role in all collaborations.

"Problems always occur. To work well with each other, we need to have collaborative plan with everyone's ownership. Secondly, we need to build trust and not allow any mistrust to happen." – Nr 27, international organisation.

"We also work closely and indirectly with selected institutions which have the most power." – Nr 3, governmental organisation.

Obstacles identified were lack of funding or long-term sustainability of the collaboration and its activities, with politics on sub-national and national level could mean unfavourable conditions for a collaboration or simply competing priorities or work of the stakeholders in the collaboration. There could also be a sense of a lack of accountability towards each other or the thought beneficiaries, with sometime faltering commitment to work together, lack of transparency of funds or efforts, and difficulty of attributing failures or successes.

"For instance if we are looking among 25 sub national civil society working group at the provincial/municipal level, there was only 50% who were active. Among these half, only 20 to 30 % who were very active in fulfilment of their collaborative work." – Nr 20, non-governmental organisation.

DISCUSSION

In this study, we found that the adoption of the SDGs led to an increased perceived possibility for action and higher ambitions for child health, perpetuating child health as a multisectoral issue. Further, there seem to be a gap between the desired step-by-step theory of conducting multisectoral collaboration and the real-world complexities of conducting such collaborations for child health in

For peer review only - http://bmjopen.bmj.com/site/about/guidelines.xhtml

Cambodia. This is the first study to provide in-country insights that can be transferable on multisectoral collaborations for child health, overcoming some of the key methodological gaps noted by Glandon et al.[31] including describing power dynamics, type of governance arrangements and a diversity of stakeholder experiences.

The expanded view of child health and higher ambition for children to thrive led to a more compelling case for multisectoral collaborations to have a collaborative advantage over single-sector or single-stakeholder efforts. The 2030 Agenda and the SDGs influence social norms at a global, country, organisational and individual level.[32] The widespread knowledge of the overarching ambition and content of the SDGs in our study serve to exemplify the notion of universality of the 2030 Agenda, and the normative significance of universality in a country context.[33] Further, the perceived high ambition of the SDGs, the diversity of topics covered in the SDGs and their interlinked nature might shift norms to be more favourable towards multisectoral collaboration, in line with Huxam´s theory of collaboration advantage.[34] Placing children firmly in the centre of the SDGs in Cambodia might also allow for a re-vitalization of action and enable policy makers and practitioners to utilise the interlinkages within the SDGs to build multisectoral collaboration for child health.[35,36]

Multisectoral collaborations depicted by the participants in this study showcase that there is often no linear process but rather ongoing non-linear flow of activities that intentionally lead to a multisectoral collaboration. The rational logic of inquiry theory whereby one step lead to the next one until a decision is made and action is implemented and evaluated originally proposed by Dewey[37] were perceived by the participants to be the desired theory or process of collaboration. However as showcased by Kuruvilla and Dorstoewitz[38] previously, the collaborations described somewhat mimic the multisectoral collaboration model[20] which rests on dynamic networks and changing contexts. In our study, participants singled out funding as an enabler and obstacle as well as a significant source of power in multisectoral collaborations. As noted by Rasanathan et al.[39] if multisectoral collaborations for health are to succeed appropriate financing systems that incentivise these collaborations must be in place, and the multisectoral monitoring and evaluation mechanisms allow for accountability. Conflicting perspectives between stakeholders, particularly government and non-governmental stakeholders, has been documented in Cambodia[25,26,40,41] and in other settings [42,43]. In our study there was a difference between interviewees from governmental organisations versus those from non-governmental particularly concerning the commitment and ability of the government to support and participate in multisectoral collaborations for child health. Although exploring this potential conflict was not the aim of this study, the emphasis of the participants on explicit and implicit territory feelings, hierarchies and power dynamics at a national and sub-national level in Cambodia strengthen the need to include these concepts in collaborative theory and when designing multisectoral collaborations.[44,45]

Our limitations include that the purposive sampling led to selection bias in the recruitment of participants. As illustrated in Table 1, the interviewees were slightly unbalanced in terms of gender and expertise in SDG areas. Participants were asked to reflect on one or two multisectoral collaborations to inform the answers to the questions in the interview, they might have had a positive

For peer review only - http://bmjopen.bmj.com/site/about/guidelines.xhtml

recall bias, only including those that were successful. Given the critical assessment of the multisectoral collaborations apparent in the interviews this seem negligible, however. Lastly, intra-personal dynamics between the interviewer and the interviewee might affect the answers and follow-up questions. In our study, the interviews were conducted by SS and TC, both representing academic institutions and being knowledgeable of qualitative research methods and the political landscape of organisations in Cambodia, ideally enabling both government and non-government stakeholders to express views and perceptions freely while adding credibility to the results. Although some of the findings in this study might reflect the unique Cambodia context, we believe that overall themes and conclusions are transferable to other middle-income countries and similar settings, adding valuable evidence on how stakeholders view multisectoral collaborations in general and specifically for child health. The study was designed to accomplish high information power across the five dimensions of information power,[22] however, with a broad research question and cross-case analysis the sample size was deemed to have to be relatively large to reach satisfactory information power and theoretical saturation. Information power was further increased by use of dense sampling method (purposive and specific), applied theory in the form of established frameworks for multisectoral collaborations, and high-quality dialogue in the interviews allowing for in-depth diverse multistakeholder perspectives.

CONCLUSION

We found that stakeholders in Cambodia perceived the SDGs to inspire an expanded view on child health that enabled change and promoted multisectoral collaboration. Interviewees experienced a gap between the desired theory of conducting multisectoral collaborations for child health and the real-world complexities of engaging in such an endeavour. The findings from this in-depth study can inform policy makers and practitioners who wish to encourage and take advantage of multisectoral collaborations for accelerating the work towards achieving better health in general and child health specifically the era of the SDGs.

Contributors

TA, HMA and DH conceived and designed the study. TC and SS contributed to the study design and conducted the data collection. DH analysed the data together with TC, SS and HMA. DH wrote the first draft of the manuscript to which all authors (DH, TC, SS, HN SK, HMA and TA) provided critical contributions. All authors read and approved the final manuscript.

Funding

The work was supported by the Swedish Research Council (2018-03609). The funding organisation were not involved in the manuscript's writing or the decision to submit it for publication.

Competing interests

None declared.

Patient and public involvement

For peer review only - http://bmjopen.bmj.com/site/about/guidelines.xhtml

No patients or public representative were directly involved in the design, conduct or reporting of this study. The findings will be disseminated and discussed with involved stakeholders. A reflexivity statement can be found in the **Supplementary Material 2.**

Patient consent for publication

Not applicable.

Ethics approval

The study received ethical approval from the National Ethics Committee for Health Research in Cambodia (NECHR-023) and was exempt from ethical review from the Swedish Ethical Review Authority (Dnr 2022-00424-01). Informed consent was obtained from all participants before inclusion in the study.

Data availability statement

No data is available. This is a qualitative study of a relatively small sample population in Cambodia. Making the dataset publicly available could potentially breach the privacy that was promised to participants when they agreed to take part and the ethical approvals granted. Therefore, the authors will not make the full transcripts available to a wider audience.

For peer review only

References

1 Independent Accountability Panel. Caught in the COIVD-19 storm: women' s , children' s , and adolescents ' health in the context of UHC and the SDGs. Geneva: 2020. https://iapewec.org/wp-content/uploads/2020/12/IAP-2020-Report_web-sp.pdf

2 United Nations. The Sustainable Development Goals Report. New York: 2022. https://unstats.un.org/sdgs/report/2022/The-Sustainable-Development-Goals-Report-2022.pdf

3 Marmot M. Achieving health equity: from root causes to fair outcomes. *Lancet* 2007;**370**:1153–63. doi:10.1016/S0140

4 Bishai DM, Cohen R, Alfonso YN, *et al.* Factors Contributing to Maternal and Child Mortality Reductions in 146 Low-and Middle- Income Countries between 1990 and 2010. *PLoS One* 2016;**11**:e0144908. doi:10.1371/journal.pone.0144908

5 Every Woman Every Child. The global strategy for women´s children´s and adolescents` health (2016-2030). New York: 2015.

6 Alfvén T, Dahlstrand J, Humphreys D, *et al.* Placing children at the center of the Sustainable Development Report: A SIGHT – Swedish Society of Medicine Road Map on Global Child Health. Stockholm: 2019.

7 Lawn JE, Rohde J, Rifkin S, *et al.* Alma-Ata 30 years on: revolutionary, relevant, and time to revitalise. *Lancet (British Ed* 2008;**372**:917–27. doi:http://dx.doi.org/10.1016/S0140-6736(08)61402-6

8 Organization WH. Time to deliver: report of the WHO Independent High-Level Commission on Noncommunicable Diseases. Geneva: 2018.

For peer review only - http://bmjopen.bmj.com/site/about/guidelines.xhtml

9 Rasanathan K, Damji N, Atsbeha T, *et al.* Ensuring multisectoral action on the determinants of reproductive, maternal, newborn, child, and adolescent health in the post-2015 era. *BMJ* 2015;**351**:h4213. doi:10.1136/bmj.h4213

10 Levinson FJ, Balarajan Y, Marini A. Addressing malnutrition multisectorally. What have we learned from recent international experience? New York: 2013.

11 Lo S, Das P, Horton R. A good start in life will ensure a sustainable future for all. *Lancet* 2017;**389**:8–9. doi:10.1016/S0140-6736(16)31774-3

12 Neuman MJ, Devercelli AE. What matters most for early childhood development? A framework paper. Systems Approach for Better Education Results (SABER) working paper series. Washington: 2013.

13 Blomstedt Y, Bhutta ZA, Dahlstrand J, *et al.* Partnerships for child health: capitalising on links between the sustainable development goals. *BMJ* 2018;**360**:k125. doi:10.1136/BMJ.K125

14 Kuruvilla S, Schweitzer J, Bishai D, *et al.* Policy and Practice: Success factors for reducing maternal and child mortality. *Bull World Heal Organ* 2014;**92**:533–44. doi:10.2471/BLT.14.138131

15 Ahmed SM, Rawal LB, Chowdhury SA, *et al.* Cross-country analysis of strategies for achieving progress towards global goals for women's and children's health. *Bull World Health Organ* 2016;**94**:351–61. doi:10.2471/BLT.15.168450

16 UNICEF. Child Poverty in Cambodia Summary Report. Phnom Penh: 2018.

17 Kaba MW, Baesel K, Poch B, *et al.* IDPoor: a poverty identification programme that enables collaboration across sectors for maternal and child health in Cambodia. *BMJ* 2018;**363**:k4698. doi:10.1136/bmj.k4698

18 UNICEF. Reducing Stunting in Children Under Five Years of Age: A Comprehensive Evaluation of UNICEF's Strategies and Programme Performance – Cambodia Country Case Study. New York: 2017.

19 Royal Government of Cambodia. Cambodian Sustainable Development Goals (CSDGs) Framework (2016-2030). Phnom Penh: 2018.

20 Kuruvilla S, Hinton R, Boerma T, *et al.* Business not as usual: how multisectoral collaboration can promote transformative change for health and sustainable development. *BMJ* 2018;**363**:k4771. doi:10.1136/bmj.k4771

21 Tong A, Sainsbury P, Craig J. Consolidated criteria for reporting qualitative research: A 32-item checklist for interviews and focus groups. *Int J Qual Heal Care* 2018;**19**:349–57.

22 Malterud K, Siersma VD, Guassora AD. Sample Size in Qualitative Interview Studies: Guided by Information Power. *Qual Health Res* 2016;**26**:1753–60. doi:10.1177/1049732315617444

23 Bertelsmann Stiftung. BTI 2022 Country Report — Cambodia. 2022.

24 Royal Government of Cambodia. National Program for Sub-National Democratic Development. Phnom Penh: 2010.

25 Un K. *Cambodia: Return to Authoritarianism*. Cambridge University Press 2019.

26 Frewer T. Doing NGO Work: the politics of being 'civil society' and promoting 'good governance' in Cambodia. *Aust Geogr* 2013;**44**:97-114,. doi:10.1080/00049182.2013.765350

27 International Council for Science. A guide to SDG interactions: from science to

For peer review only - http://bmjopen.bmj.com/site/about/guidelines.xhtml

implementation. Paris: 2017.

28 World Health Organization. Health in all policies: Helsinki statement. Framework for country action. Geneva: 2014.

29 Gale N, Heath G, Cameron E, *et al.* Using the framework method for the analysis of qualitative data in multi-disciplinary health research. *BMC Med Res Methodol 2013,* 2013;**13**:260–1.

30 Malterud K. Theory and interpretation in qualitative studies from general practice: Why and how? *Scand J Public Health* 2016;**44**:120–9. doi:10.1177/1403494815621181

31 Glandon D, Mondal S, Okeyo I, *et al.* Methodological gaps and opportunities for studying multisectoral collaboration for health in low- and middle-income countries. *Health Policy Plan* 2019;**34**:ii7–17. doi:10.1093/heapol/czz116

32 Schmieg G, Meyer E, Schrickel I, *et al.* Modeling normativity in sustainability: a comparison of the sustainable development goals, the Paris agreement, and the papal encyclical. *Sustain Sci* 2018;**13**:785–96. doi:10.1007/s11625-017-0504-7

33 Long G. The idea of universality in the sustainable development goals. *Ethics Int Aff* 2015;**29**:203–22. doi:10.1017/S0892679415000076

34 Huxham C. Theorizing collaboration practice. *Public Manag Rev* 2003;**5**:401–23. doi:10.1080/1471903032000146964

35 Alfvén T, Dahlstrand J, Humphreys D, *et al.* Placing children and adolescents at the centre of the Sustainable Development Goals will deliver for current and future generations. *Glob Health Action* 2019;**12**:1670015. doi:10.1080/16549716.2019.1670015

36 Clark H, Coll-seck AM, Banerjee A, *et al.* The Lancet Commissions A future for the world ’ s children ? A WHO – UNICEF – Lancet Commission. *Lancet* 2020;**395**:605–58. doi:10.1016/S0140-6736(19)32540-1

37 Dewey J. *Logic: The Theory of Inquiry*. New York: : Holt, Rinehart and Winston 1938.

38 Kuruvilla S, Dorstewitz P. There is no ‘point’ in decision-making: A model of transactive rationality for public policy and administration. *Policy Sci* 2010;**43**:263–87. doi:10.1007/s11077-009-9098-y

39 Rasanathan K, Atkins V, Mwansambo C, *et al.* Governing multisectoral action for health in low-income and middleincome countries: An agenda for the way forward. *BMJ Glob Heal* 2018;**3**:1–6. doi:10.1136/bmjgh-2018-000890

40 Schröder P, Young S. The Implications of Closing Civic Space for Sustainable Development in Cambodia. Sussex: 2019.

41 Norén-Nilsson A, Bourdier F. Introduction: Social Movements in Cambodia. *J Curr Southeast Asian Aff* 2019;**38**:3–9. doi:10.1177/1868103419848192

42 Rajabi M, Ebrahimi P, Aryankhesal A. Collaboration between the government and nongovernmental organizations in providing health-care services: A systematic review of challenges. *J Educ Health Promot* 2021;**10**:1–9. doi:10.4103/jehp.jehp

43 Ejaz I, Shaikh B, Rizvi N. NGOs and government partnership for health systems strengthening:A qualitative study presenting viewpoints of government, NGOs and donors in Pakistan. *BMC Health Serv Res* 2011;**11**:122. doi:https://doi.org/10.1186/1472-6963-11-122

44 Faul M V. Multi-sectoral partnerships and power. Geneva: 2015.

For peer review only - http://bmjopen.bmj.com/site/about/guidelines.xhtml

45 Purdy JM. Power in Collaborative Governance. *Public Adm Rev* 2012;**72**:409–17. doi:10.111/j.1540-6210.2012.02525.x.A

For peer review only

Supplementary Material 1

Table of Contents

Interview Guide 2

Consolidated criteria for reporting qualitative studies (COREQ): 32-item checklist 6

Full coding tables 9

For peer review only

For peer review only - http://bmjopen.bmj.com/site/about/guidelines.xhtml

Interview Guide

1. Background information. *Please mark and fill in the following questions on this sheet*

1. How many years have you worked: ________

2. Sex ☐ Female ☐ Male

3. Education

Highest degree:________________________________

Topic of degree:________________________________

4. Work experience

Current employment

Organization:________________________ **Role/Position:**________________________

Work experience from the following sectors (represented by Cambodia Sustainable Development Goals) - Multiple Answers

**Sectors**	**Rural**	**Urban**	**Sectors**	**Rural**	**Urban**
1. Poverty/social protection	☐	☐	10. Income inequality	☐	☐
2. Food and nutrition	☐	☐	11. Sustainable cities/communities/urban planning	☐	☐
3. Health and well-being	☐	☐	12. Safe consumption and production	☐	☐
4. Education	☐	☐	13. Climate change	☐	☐
5. Gender equality	☐	☐	14. Life bellow water/ocean	☐	☐
6. Water and sanitation	☐	☐	15. Life on land/natural resources	☐	☐
7. Energy	☐	☐	16. Institutional strengthening/anti corruption/legislation	☐	☐
8. Labor market, financial sector	☐	☐	17. Partnerships/collaborative networks	☐	☐
9. Industry and infrastructure	☐	☐	18. Cambodia Mine/ERW free	☐	☐

For peer review only

For peer review only - http://bmjopen.bmj.com/site/about/guidelines.xhtml

Other:	☐	☐
**Have you worked related to child health (both health and non-health sector)?** ☐ Yes ☐ No<br><br>**If yes, please describe shortly in what way:**		

Thank you for providing telling us your background, we would now like to know a little bit more about your views on the topics of Sustainable Development, child health and multisectoral work in general.

2. Background: Sustainable Development Goals, child health and multisectoral work

The 2030 Agenda and the 17 Sustainable Development Goals is a framework with a comprehensive set of goals adopted by the UN in 2015 for all countries to end poverty, protect the planet and ensure prosperity for all. These have been adapted to the Cambodia Sustainable Goals.

2.1. What were your first thoughts/opinion when the 2030 Agenda and the Sustainable Development Goals were launched?

2.1.2. What have your organization done now in relation to the Sustainable Development Goals in Cambodia compared to what you did with the Millennium Development Goals before and? (*mode of work rather than specific activities*)

2.2. Could you please describe what you would say child health includes?

2.2.1 What age? What to you include in the term "health" when it comes to children?

2.2.2 Are there any particular aspects of the health of children that you think the health system need to take special consideration to?

2.3. How do you think actors in Cambodia contribute in supporting child health to implement the Cambodia Sustainable Development Goals?

2.4. What are the linkages/connections between child health and non-health sectors (as represented by the Cambodia Sustainable Development Goals)?

2.4.1 Please provide examples of such linkages from your current/former work or what you have observe in the society?

2.4.2 Many sectors and activities can influence child health. Which sectors do you think are most relevant for child health?

Thank you for providing your views on these topics, now we would like to ask you some further questions on the multisectoral work around child health that you have experience from. Please

For peer review only - http://bmjopen.bmj.com/site/about/guidelines.xhtml

think of a collaboration specifically, or generally if you have experience from many different, between two or more sectors that had the explicit goal to in some way increase child health and well-being.

3. Multisectoral collaboration for child health

Based on the Multisectoral collaborative model and the Health in all policies approach.

i) Drive change/Establishing the need for multisectoral work

3.1. Which organizations are your key stakeholders to work in promoting child health?

3.1. What was it that made the partners in the collaboration identify the need for multisectoral work? How did it begin?

ii) Defining the problem and constraints

3.2. How was the above-mentioned child health need identified, defined or framed?

iii) Design of the collaboration/Planned framed action & Supportive structures and policies

3.3. Was there a planning process of how to conduct the multisectoral work?

3.4. Which stakeholders were involved in planning?

3.5. How was the work of collaboration designed to be carried out?

3.5.1 How was the coordination organized?

3.5.2 How was the collaboration implemented?

3.5.3 How was the work of the collaboration financed or mobilized?

3.5. How do you think the multisectoral work were actually implemented compared to the plan?

3.6. Where there any supportive structures or policies in place that enabled the work to be conducted?

iv) Capture success / Monitoring and evaluation

3.7. How was the multisectoral work monitored and evaluated?

3.7.1 Was there any key indicators or markers of success monitored?

3.7.2 How was the success or failure attributed to between the partners in the collaboration?

V) Relate / Facilitate assessment and engagement & Build capacity

3.8. How did the relationship between the partners evolve during the multisectoral work?

For peer review only - http://bmjopen.bmj.com/site/about/guidelines.xhtml

3.9. Did the collaborating partners make any effort to improve their relationship?

3.10. Where there any efforts to engage with a wider group of actors or the public in the work?

4. Where there any type of capacity building activities included in the collaboration?

Final questions

5.1. What are your suggestions and recommendations in order to improve multisectoral collaboration to promote child health?

5.2. Are there any end points you want to add on any of the topics touched upon today or that we have not spoken about?

For peer review only

For peer review only - http://bmjopen.bmj.com/site/about/guidelines.xhtml

Consolidated criteria for reporting qualitative studies (COREQ): 32-item checklist

No.	Item	Description	Page
Domain 1: Research team and reflexivity			
Personal characteristics			
1	Interviewer/facilitator	SS and TC conducted the interviews	4
2	Credentials	DH has a MD, SS has PhD, TC has MD and has a Master of Arts in Health Social Sciences, HN has a PhD, KR has a PhD, HMA has a PhD and TA has a PhD.	1
3	Occupation	DH was a PhD candidate, SS was a lecturer at the Royal University of Phnom Penh, TC was a program manager at Malaria Consortium Cambodia.	1
4	Gender	All interviewers were male.	NA
5	Experience and training	The researchers (SS and TC) had extensive experience of qualitative interviews from previous research in Cambodia.	16
Relationship with participants			
6	Relationship established	No relationship was established prior to study commencement.	16
7	Participant knowledge of the interviewer	In some instances, the participant recognized the interviewer from attending similar events/workshops/seminars but in general the participants did not know the interviewers. They did not know the personal goals or reasons for doing the research for the individual interviewer.	16
8	Interviewer characteristics	The interviewers were all interested in the topic in general and had expertise in child health in Cambodia.	4
Domain 2: Study design			

For peer review only - http://bmjopen.bmj.com/site/about/guidelines.xhtml

Theoretical framework			
9	Methodological orientation and theory	The methodological orientation of the study is content analysis, specifically framework analysis.	4
Participant selection			
10	Sampling	Participants were purposively selected based on predefined criteria of having expertise in child health or being from a non-health sector (for example water and sanitation, agriculture, infrastructure etc.) but with implementation knowledge of how that sector interacts with other sectors in Cambodia.	4
11	Method of approach	Participants were approached via email and telephone.	NA
12	Sample size	29 participated in the interviews.	4
13	Non-participation	No participants refused or dropped out.	4
Setting			
14	Setting of data collection	The interviews took place either virtually (over online meeting) or face to face, at a time and place convenient of the participant.	4
15	Presence of non-participants	The were no non-participants present during the interviews.	4
16	Description of sample	The description of the sample can be seen in Table 1 in the article.	5
Data collection			
17	Interview guide	This is provided in the supplementary material 1. The interview guide was piloted before the study began.	4-5
18	Repeat inverviews	No repeat interviews were held.	NA
19	Audio/visual recording	Audio recording was used to collect the data.	4
20	Field notes	No field notes were taken.	4
21	Duration	The duration of the interviews ranged from 45 minutes to 1 hr and 15 minutes	NA

For peer review only - http://bmjopen.bmj.com/site/about/guidelines.xhtml

22	Data saturation	Is discussed with regards to information power in the article.	16
23	Transcripts returned	Transcripts were not returned to participants.	NA
Domain 3: Analysis and findings			
Data analysis			
24	Number of data coders	DH coded the data	8
25	Description of the coding tree	Is presented in Table 2 in the manuscript and supplementary material 1.	9
26	Derivation of themes	The themes were derived from the data.	8
27	Software	Nvivo software were used for the coding.	8
28	Participant checking	The participants did not provide feedback on the findings.	NA
Reporting			
29	Quotations presented	Quotations presented with each paragraph, trying to illustrate the main points.	10-14
30	Data and findings consistent	The data and findings were cross-checked multiple times, ensuring consistency.	16
31	Clarity of major themes	Outlined in result table and in clear headings in the result section.	9-14
32	Clarity of minor themes	Outlined in result table and in clear headings in the result section.	9-14

For peer review only - http://bmjopen.bmj.com/site/about/guidelines.xhtml

Full coding tables

Main themes and findings - full coding tables

**Theme**	SDGs and expanded view on child health enable change			
**Sub-themes**	Possibility for action due to SDGs			
**Categories**	Government commitment to and leadership of SDGs	SDGs provide a common vision and guide		Discrepancy between ambition and actual work
**Subcategories**		More detailed than MDGs	Showcase that health is a multisectoral issue	
**Codes**	Adoption and change of national plans and policies<br><br>No change in government as leaders of the goals<br><br>SDG implementation depends on alignment to government	Provide a clear set of goals<br><br>Provide a roadmap or guide<br><br>More detailed<br><br>More complex reflecting actual conditions	Illustrate that health is a multisectoral issue<br><br>SDGs reflecting actual conditions with regards to health	SDGs too complex, impossible to succeed<br><br>High ambition not matched with resources/work committed

For peer review only

Main themes and findings - full coding tables, continued.

**Themes**	SDGs and expanded view on child health enable change					
**Sub-themes**	Higher ambitions for child health, a multisectoral area at heart					
**Categories**	Definition of child health		Child health linkages across sectors			
**Sub-categories**	Child age under 18 years	A focus on not only health but well-being	Child health by definition a multisectoral issue	Education and schooling	Nutrition	General societal conditions
**Codes**	General view and legally a child is a person under 18 years of age	Physical and mental health equally important<br><br>Good nutrition and absence of disease	All SDGs important for child health<br><br>The linkages between sectors and child health cannot be divided	Education as most formative experience<br><br>School important physical place for linkages<br><br>Early child development key	Nutrition and functioning agricultural sector as basis for child growth<br><br>Commercial interests conflicts with good child nutrition	Physical safety and hygiene environment<br><br>Economic development of country<br><br>Social protection systems

For peer review only - http://bmjopen.bmj.com/site/about/guidelines.xhtml

Main themes and findings - full coding tables, continued.

**Theme**	SDGs and expanded view on child health enable change				
**Sub-themes**	Higher ambitions for child health, a multisectoral area at heart				
**Categories**	Aspects of the health system and actors unique to children			Special considerations for children	
**Sub-categories**	Responsibility of family and community	Influence of other actors	Key aspects of health system for improving child health	Life course approach	Enabling the child to thrive
**Codes**	Parents and family are the primary caretaker<br><br>Information and health literacy key undertaking<br><br>Social determinants of family dictates child health to large extent	Government overarching leader and supporter of child health<br><br>International organizations influence organizations in country<br><br>Commercial interests of private sector	Lack of focus on preventive child health measures<br><br>Need to improve quality and equity<br><br>Difference between rural and urban areas	Prenatal services important for child health<br><br>Children have different needs at different ages	A focus on child growth<br><br>Holistic approach<br><br>Acknowledging child rights

For peer review only

For peer review only - http://bmjopen.bmj.com/site/about/guidelines.xhtml

Main themes and findings - full coding tables, continued.

**Theme**	Gap between theory and real world complexities					
**Sub-themes**	Linear process of collaboration					
**Categories**	Actors and topics			Identifying and framing problem		
**Sub-categories**	Broad variety of actors	Territory feelings	Collaborations focused on non-health aspects	Top-down approach	Bottom-up approach	Framing of problem
**Codes**	Government as natural leader<br><br>Civil society networks<br><br>External donors emphasize importance<br><br>Many different actors collaborating	There exist strict boundaries between actors<br><br>Competition between actors for funding<br><br>Skeptical view of government and NGO and vice versa	Focused on preventive issues<br><br>Collaboration indirectly see effect on child health<br><br>Willingness to connect to child health	Government or ministries identifies need<br><br>National policy or development plan<br><br>International agenda or external funding opportunities<br><br>From own organizational strategy or values	Listening to stakeholders in community or on sub-national level<br><br>Routine data or findings from actual situation on the ground<br><br>Reliable data not always present	Research as a way of narrowing problem<br><br>Involving many actors in collective process<br><br>Detailed problem statement

For peer review only - http://bmjopen.bmj.com/site/about/guidelines.xhtml

Main themes and findings - full coding tables, continued.

**Theme**	Gap between theory and real world complexities						
**Sub-themes**	Linear process of collaboration						
**Categories**	Planning			Coordination			
**Sub-categories**	Complex, detailed, resource constraining process	Capacity assessment key	Prioritization depending on context	Varied methods of coordination	Clear division of responsibilities	Leadership paramount	Power and hierarchies influence coordination
**Codes**	Many actors involved in planning<br><br>Sub-national and national level engaged<br><br>Technical level and strategy level<br><br>Detailed collaboration plan and outline of activities, outputs,	Technical skill and resource capacity at implementer level instrumental<br><br>Division of activities based on capacity<br><br>If not enough capacity collaboration cannot begin	Prioritization based on funding requirements<br><br>Politics and benefits of including certain actors or activities	Information sharing mechanisms<br><br>Focal points or joint committees<br><br>Regular, continuous coordination<br><br>Built on existing structures	Agreed upon plan of responsibilities<br><br>Common vision and commitment key for ease of coordination<br><br>Participation in joint coordination hard	Single organization that explicitly or implicitly lead<br><br>Structuring collaboration efforts depends on leader	Focal points for collaboration lack decision making power<br><br>Power imbalance due to government more powerful<br><br>Competing for funding between organizations

For peer review only - http://bmjopen.bmj.com/site/about/guidelines.xhtml

	and desired outcomes<br><br>Commitment and ownership implicit goals of process						

For peer review only

Main themes and findings - full coding tables, continued.

**Theme**	Gap between theory and real world complexities						
**Sub-themes**	Linear process of collaboration						
**Categories**	Implementation			Monitoring and evaluation			
**Sub-categories**	Adaptability to change	Geographical and administrative level	Follows from planning and coordination	Detailed but depends on funding	Hard to move beyond outputs	Integral to the collaboration	Responsibility for M&E varies
**Codes**	Implementation does not follow plan<br><br>Funding changes requires change of plan<br><br>Government involvement lead to less flexibility<br><br>Covid-19 disruption	Focus on implementing organizations or participations<br><br>Added complexity for actual implementation<br><br>National level collaboration, sub-national implement<br><br>Sub-national own system of priorities,	Implementation mirrors previous collaborative efforts<br><br>Reduction in parallel work and efficient implementation<br><br>Takes time and resources to implement, need to be considered before start	Funding source and resources key for allowing M&E<br><br>M&E include detailed indicators<br><br>Government or external donor relies heavily on M&E for decisions	Discrepancy in M&E between stakeholders<br><br>Particularly hard to attribute success or failures<br><br>Quantitative indicators more favorable	Learning from failure<br><br>M&E seen as opportunity to learn and improve<br><br>Successes can build momentum, secure resources<br><br>Serves as main accountability mechanism	Internal or external evaluation depending on context and resources<br><br>One stakeholder monitors activities<br><br>Joint monitoring of activities

For peer review only - http://bmjopen.bmj.com/site/about/guidelines.xhtml

		relationships and focus					

For peer review only - http://bmjopen.bmj.com/site/about/guidelines.xhtml

Main themes and findings - full coding tables, continued.

**Theme**	Gap between theory and real world complexities						
**Sub-themes**	Linear process of collaboration		Real-world complexities shaping the collaboration				
**Categories**	Dissemination		Funding		Relationships		
**Sub-categories**	Information spreading	Recognition	Call for more funding	Funding as a source of power	Facilitate or hamper collaboration	Actively building relationships	Relationships as an outcome
**Codes**	Engage the public and stakeholders<br><br>Increase awareness	Engaging national-level government<br><br>Gain international reputation	Budget greatest limitation to collaboration<br><br>Not enough government/national funding<br><br>Funding sources varies<br><br>If government funding more sustainable	External donors agenda decide activities<br><br>If funding from government they have last say<br><br>Leadership often based on funding<br><br>Ministry of economy key stakeholder	Tensions between NGOs and government evident<br><br>Conflicts within government or NGO networks<br><br>Common understanding and relationships increase coordination	Continuous relationship building<br><br>Efforts by stakeholders to build relationships	Over time relationships built through coordination meetings and implementation<br><br>Evolve between key focal points<br><br>Mutual understanding lead to trust and confidence

For peer review only - http://bmjopen.bmj.com/site/about/guidelines.xhtml

				Decide design and coordination of collaboration			

For peer review only

Main themes and findings - full coding tables, continued.

**Theme**	Gap between theory and real world complexities									
**Sub-themes**	Real-world complexities shaping the collaboration					Critically assessing collaborations				
**Categories**	Enabling environment		Capacity building			Success factors			Obstacles	
**Sub-categories**	Policies	Government	Actual method depends on collaboration	Key for sustainability	Demands resources	Clear responsibilities	Common vision and understanding	Secure buy in	Real world complexities	Lack of accountability
**Codes**	International agenda facilitate work<br><br>Sub-national plans for development<br><br>National CSDG roadmap and other national plans	Active and collaborative government ministries<br><br>Existing multisectoral ministerial committees	In person technical capacity building<br><br>Natural reciprocal<br><br>Effort to include capacity building<br><br>Capacity building according to administrative	Learning and incorporating changes<br><br>Integral part of collaboration itself, one of main benefits<br><br>Building capacity with implementors or sub/national level lead to sustainability	Capacity building takes time<br><br>Capacity building limited by funding	Agreement on division of activities<br><br>Leadership from all<br><br>Functioning M&E	Learning continuously<br><br>Open sharing and discussion<br><br>Benefits and goals explicit	Engage stakeholders from beginning<br><br>Government and sustainable funding<br><br>Commitment from all<br><br>Relationship and capacity building	Lack of funding, sustainability<br><br>Politics on sub-national and national level<br><br>Competing priorities and work	No commitment to work together<br><br>Lack of transparency<br><br>Difficulty of attributing failures or successes

For peer review only - http://bmjopen.bmj.com/site/about/guidelines.xhtml

			and geographical level							

For peer review only - http://bmjopen.bmj.com/site/about/guidelines.xhtml

Supplementary Material 2. Reflexivity Statement

Study conceptualisation:

1. How does this study address local research and policy priorities?

This study is a part of an effort to provide country specific knowledge of multisectoral collaborations in Cambodia, a key knowledge gap identified by stakeholders and academia. The government and other organizations are actively engaging in multisectoral collaborations, and understanding how the function in practice is a key priority.

2. How were local researchers involved in study design?

The local researchers (SS and TC) were engaged in the overall design of the study and particularly the identification and recruitment of participants as well as development of the interview guides and data collection. They were core members of the study team.

Research management:

1. How has funding been used to support the local research team(s)?

The study was funded through the Swedish Research Council (2018-03609) with the majority of funding dedicated to country study activities and local research colleagues (SS and TC).

Data acquisition and analysis:

1. How are research staff who conducted data collection acknowledged?

The researchers who conducted data collection met the authorship criteria and are hence acknowledged as co-authors of the study.

2. How have members of the research partnership been provided with access to study data?

All members of the research team, including SS and TC, had full access to the data.

3. How were data used to develop analytical skills within the partnership?

The qualitative data analysis was conducted by DH with input and training of DH, SS and TC by a qualitative research expert (HMA).

Data interpretation:

1. How have research partners collaborated in interpreting study data?

The results from the study were continuously discussed with the local research colleagues (SS and TC) who contributed significantly to the interpretation of the results.

Drafting and revising for intellectual content:

1. How were research partners supported to develop writing skills?

Most of the writing of the manuscript was done by DH, however local research colleagues (SS and TC) provided crucial input.

For peer review only

2. How will research products be shared to address local needs?
The results from the study will be disseminated widely to an international and national audience, including a dissemination seminar with relevant country stakeholders.

Authorship:

1. How is the leadership, contribution and ownership of this work by LMIC researchers recognized within the authorship?
The local researchers (SS and TC) authors 2-3, recognizing their crucial hands-on contribution to the study.

2. How have early career researchers across the partnership been included within the authorship team?
The first author is a PhD student (although not from a LMIC), SS and TC are recognized experienced researchers.

3. How has gender balance been addressed within the authorship?
Out of the seven authors, four are male (DH, SS, TC and TA) while three (HN, SK and HMA) are female. The preponderance for male authors is weighted against the critical study design and interpretation by HN and SK while HMA is a world-leading qualitative expert.

Training:

1. How has the project contributed to training of LMIC researchers?
The LMIC researchers (SS and TC) are experienced qualitative researchers, however within this study all authors gained refresher trainings and developed their qualitative analytical skills and knowledge of framework method analysis by HMA (qualitative expert).

Infrastructure:

1. How has the project contributed to improvements in local infrastructure?
No direct benefit in local infrastructure has come from this qualitative study, however the findings of the study can help to conceptualize and form partnerships across sectors that can lead to improvements in infrastructure.

Governance:
1. What safeguarding procedures were used to protect local study participants and researchers?
The study conforms to the Helsinki declaration and followed the ethical and practical guidelines stipulated by the National Ethics Committee for Health Research in Cambodia regarding the safety of researchers and participants.

For peer review only

Sustainable development goals and multisectoral collaborations for child health in Cambodia: a qualitative interview study with key child health stakeholders

Consolidated criteria for reporting qualitative studies (COREQ): 32-item checklist

No.	Item	Description	Page
Domain 1: Research team and reflexivity			
Personal characteristics			
1	Interviewer/facilitator	SS and TC conducted the interviews	4
2	Credentials	DH has a MD, SS has PhD, TC has MD and has a Master of Arts in Health Social Sciences, HN has a PhD, KR has a PhD, HMA has a PhD and TA has a PhD.	1
3	Occupation	DH was a PhD candidate, SS was a lecturer at the Royal University of Phnom Penh, TC was a program manager at Malaria Consortium Cambodia.	1
4	Gender	All interviewers were male.	NA
5	Experience and training	The researchers (SS and TC) had extensive experience of qualitative interviews from previous research in Cambodia.	16
Relationship with participants			
6	Relationship established	No relationship was established prior to study commencement.	16

For peer review only - http://bmjopen.bmj.com/site/about/guidelines.xhtml

7	Participant knowledge of the interviewer	In some instances, the participant recognized the interviewer from attending similar events/workshops/seminars but in general the participants did not know the interviewers. They did not know the personal goals or reasons for doing the research for the individual interviewer.	16
8	Interviewer characteristics	The interviewers were all interested in the topic in general and had expertise in child health in Cambodia.	4
Domain 2: Study design			
Theoretical framework			
9	Methodological orientation and theory	The methodological orientation of the study is content analysis, specifically framework analysis.	4
Participant selection			
10	Sampling	Participants were purposively selected based on predefined criteria of having expertise in child health or being from a non-health sector (for example water and sanitation, agriculture, infrastructure etc.) but with implementation knowledge of how that sector interacts with other sectors in Cambodia.	4
11	Method of approach	Participants were approached via email and telephone.	NA
12	Sample size	29 participated in the interviews.	4
13	Non-participation	No participants refused or dropped out.	4
Setting			
14	Setting of data collection	The interviews took place either virtually (over online meeting) or face to face, at a time and place convenient of the participant.	4
15	Presence of non-participants	The were no non-participants present during the interviews.	4
16	Description of sample	The description of the sample can be seen in Table 1 in the article.	5
Data collection			

For peer review only - http://bmjopen.bmj.com/site/about/guidelines.xhtml

17	Interview guide	This is provided in the supplementary material 1. The interview guide was piloted before the study began.	4-5
18	Repeat inverviews	No repeat interviews were held.	NA
19	Audio/visual recording	Audio recording was used to collect the data.	4
20	Field notes	No field notes were taken.	4
21	Duration	The duration of the interviews ranged from 45 minutes to 1 hr and 15 minutes	NA
22	Data saturation	Is discussed with regards to information power in the article.	16
23	Transcripts returned	Transcripts were not returned to participants.	NA
Domain 3: Analysis and findings			
Data analysis			
24	Number of data coders	DH coded the data	8
25	Description of the coding tree	Is presented in Table 2 in the manuscript and supplementary material 1.	9
26	Derivation of themes	The themes were derived from the data.	8
27	Software	Nvivo software were used for the coding.	8
28	Participant checking	The participants did not provide feedback on the findings.	NA
Reporting			
29	Quotations presented	Quotations presented with each paragraph, trying to illustrate the main points.	10-14
30	Data and findings consistent	The data and findings were cross-checked multiple times, ensuring consistency.	16
31	Clarity of major themes	Outlined in result table and in clear headings in the result section.	9-14
32	Clarity of minor themes	Outlined in result table and in clear headings in the result section.	9-14

For peer review only - http://bmjopen.bmj.com/site/about/guidelines.xhtml

Sustainable development goals and multisectoral collaborations for child health in Cambodia: a qualitative interview study with key child health stakeholders

Journal:	*BMJ Open*
Manuscript ID	bmjopen-2023-073853.R1
Article Type:	Original research
Date Submitted by the Author:	07-Aug-2023
Complete List of Authors:	Helldén, Daniel ; Karolinska Institutet, Department of Global Public Health<br>Sok, Serey; Royal University of Phnom Penh, Research Office<br>Chea, Thy; Malaria Consortium<br>Nordenstedt , Helena; Karolinska Institute, Department of Global Public Health<br>Kuruvilla, Shyama ; WHO International<br>Alvesson, Helle; Karolinska Institutet, Department of Global Public Health<br>Alfvén, Tobias ; Karolinska Institute, Global Public Health; Sachs´ Children and Youth Hospital
**Primary Subject Heading**:	Health policy
Secondary Subject Heading:	Global health, Qualitative research, Paediatrics
Keywords:	Health policy < HEALTH SERVICES ADMINISTRATION & MANAGEMENT, Community child health < PAEDIATRICS, Organisation of health services < HEALTH SERVICES ADMINISTRATION & MANAGEMENT

I, the Submitting Author has the right to grant and does grant on behalf of all authors of the Work (as defined in the below author licence), an exclusive licence and/or a non-exclusive licence for contributions from authors who are: i) UK Crown employees; ii) where BMJ has agreed a CC-BY licence shall apply, and/or iii) in accordance with the terms applicable for US Federal Government officers or employees acting as part of their official duties; on a worldwide, perpetual, irrevocable, royalty-free basis to BMJ Publishing Group Ltd ("BMJ") its licensees and where the relevant Journal is co-owned by BMJ to the co-owners of the Journal, to publish the Work in this journal and any other BMJ products and to exploit all rights, as set out in our licence.

The Submitting Author accepts and understands that any supply made under these terms is made by BMJ to the Submitting Author unless you are acting as an employee on behalf of your employer or a postgraduate student of an affiliated institution which is paying any applicable article publishing charge ("APC") for Open Access articles. Where the Submitting Author wishes to make the Work available on an Open Access basis (and intends to pay the relevant APC), the terms of reuse of such Open Access shall be governed by a Creative Commons licence – details of these licences and which Creative Commons licence will apply to this Work are set out in our licence referred to above.

Other than as permitted in any relevant BMJ Author's Self Archiving Policies, I confirm this Work has not been accepted for publication elsewhere, is not being considered for publication elsewhere and does not duplicate material already published. I confirm all authors consent to publication of this Work and authorise the granting of this licence.

For peer review only

Title: Sustainable development goals and multisectoral collaborations for child health in Cambodia: a qualitative interview study with key child health stakeholders

Authors: Daniel Helldén[1]*, Serey Sok[2], Thy Chea[3], Helena Nordenstedt[1], Shyama Kuruvilla[4], Helle Mölsted Alvesson[1], Tobias Alfvén[1,5]

Affiliations:

[1]Department of Global Public Health, Karolinska Institutet, Stockholm, Sweden

[2]Research Office, Royal University of Phnom Penh, Phnom Penh, Cambodia

[3]Malaria Consortium, Phnom Penh, Cambodia

[4]World Health Organization, Geneva, Switzerland

[5]Sachs' Children and Youth Hospital, Stockholm, Sweden

Corresponding author details

Address: Tomtebodavägen 18 A, SE-171 77 Stockholm, Sweden

Email: daniel.hellden@ki.se

Telephone: +46732406555

Keywords: multisectoral collaboration, health policy, child health, SDGs

Number of

Tables: 2

Figures: 0

Words: 4952

For peer review only - http://bmjopen.bmj.com/site/about/guidelines.xhtml

Abstract

Objectives: Multisectoral collaboration highlighted as key in delivering on the Sustainable Development Goals (SDGs), but still little is known on how to move from rhetoric to action. Cambodia has made remarkable progress on child health over the last decades with multisectoral collaborations being a key success factor. However, it is not known how country stakeholders perceive child health in the context of the SDGs or multisectoral collaborations for child health in Cambodia.

Design, settings and participants: Through purposive sampling, we conducted semi-structured interviews with 29 key child health stakeholders from a range of government and non-governmental organisations in Cambodia. Guided by framework analysis, themes, sub-themes and categories were derived.

Results: We found that the adoption of the SDGs led to increased possibility for action and higher ambitions for child health in Cambodia, while simultaneously establishing child health as a multisectoral issue among key child stakeholders. There seem to be a discrepancy between the desired step-by-step theory of conducting multisectoral collaboration and the real-world complexities including funding and power dynamics that heavily influence the process of collaboration. Identified success factors for multisectoral collaborations included having clear responsibilities, leadership from all and trust among stakeholders while the major obstacle found was lack of sustainable funding.

Conclusion: The findings from this in-depth multistakeholder study can inform policy makers and practitioners in other countries on the theoretical and practical process as well as influencing aspects that shape multisectoral collaborations in general and for child health specifically. This is vital if multisectoral collaborations are to be successfully leveraged to accelerate the work towards achieving better child health in the era of the SDGs.

Strengths and limitations

- Using semi-structured interviews, diverse themes around the complex phenomenon of multisectoral collaboration for child health could be explored to reach high information power.

- The study included a relatively large sample of child health stakeholders at a national level with unique insights into multisectoral collaboration and knowledge of the Cambodian context.

- The sample participants interviewed is unbalanced in terms of gender and expertise in different SDG areas.

For peer review only

INTRODUCTION

Almost halfway until the United Nations Sustainable Development Goals (SDGs) are to be achieved, practitioners, experts and policy makers are trying to speed up the pace of progress on child health. This has become even more urgent with the setback of the COVID-19 pandemic which left 147 million children out of proper education, rising child labor and significantly higher rates of malnutrition and over 22 million children missing essential vaccinations.[1,2] Over the last decades it has become evident that progress made in other sectors heavily impact the possibility to make progress on child health and well-being.[3,4] Child survival is included in SDG 3 (Good health and well-being) while the broader aspects of child health and well-being is captured by many different SDGs, for instance SDG 2 (Zero hunger), SDG 4 (Quality education) and SDG 5 (Gender equality). Further, progress on child health and well-being are essential for tackling poverty and promote the development of societies.[5] Moving beyond mere child survival, there is now a larger focus on enabling children to thrive and reach their full potential.[5,6]

Multisectoral collaborations have long been seen as critical for achieving gains in health and well-being when it comes to universal health coverage, non-communicable diseases and succeeding in governing multisectoral issues going back to the World Health Organization (WHO) Constitution and the Alma Ata Declaration.[7–9] For child health a multisectoral approach to areas such as nutrition[10] and education[11,12] have been studied, however there is lack of understanding how multisectoral collaborations work out on a country level. Further, a generic analysis of the linkages between the SDGs and child health found that there are many synergies between making progress on the SDGs and accelerating progress on child health, suggesting that multisectoral collaboration could harness synergies and better handle tradeoffs between the SDGs and child health.[13]

During the Millennium Development Goal era many countries made significant gains in child health, and approximately half of the reduction in child mortality between 1990 and 2010 have been attributed to investments in sectors outside of health.[14] Cambodia was one of the fast-track countries and made significant progress including succeeded in lowering the under-five mortality from 116 to 29 deaths per 1000 live births from 1990 to 2015.[15] Many challenges persist however, with significant inequalities between rural and urban areas, lower than desired educational attainment and sub-optimal water and sanitation conditions in schools and residential areas.[16] It has been shown that multisectoral efforts, such as the ID Poor program, have been successful in reducing poverty and collaborative initiatives between non-health sectors have become a cornerstone of the maternal and child health strategy in Cambodia.[17–19]

Cambodia has managed to improve the health and well-being of children over a short period of time while utilizing collaborations across sectors to do so among other changes. However, it is not known how child health stakeholders have been influenced by the SDGs or how they theorize multisectoral collaborations, here defined as "multiple sectors and stakeholder intentionally coming together and collaborating in a managed process to achieve shared outcomes and common goals"[20], versus the actual practice of conducting such collaborations. This knowledge could inform current and future

For peer review only - http://bmjopen.bmj.com/site/about/guidelines.xhtml

multisectoral collaborations on critical theories and key success factors and obstacles when initiating and implementing such a collaboration. Hence, our aim was to understand how stakeholders in Cambodia perceive the SDGs, child health in the era of the SDGs and multisectoral collaborations for child health in Cambodia.

METHODS

Study design and setting

Guided by the The COnsolidated criteria for REporting Qualitative (COREQ) recommendations[21] and the concept of information power[22] this study utilizes semi-structured interviews to investigate how Cambodian stakeholders perceive the SDGs, the concept of child health in the era of the SDGs and multisectoral collaborations for child health in Cambodia. The country is governed primarily through the national government, which consist of the council of ministers led by the prime minister while the parliament (national assembly and senate) have legislative power. Administratively the country is divided into provinces, districts, communes and villages.[23] During the last decade, the government has incrementally favored a more decentralized approach where districts and commune government officials are given more funding and implementing power.[24] Collaboration between government and non-government stakeholders primarily occur on two levels, the national or sub-national (district or commune) level and been characterized by an increased role of the government in leading and coordinating collaborations.[25,26] The Ministry of Health and its National Maternal and Child Health Center is responsible for health services throughout Cambodia, often working in committees or technical groups with other relevant ministries and in collaboration with international and Cambodian non-governmental organisations. At the sub-national government level, provincial health departments and operational health districts lead the implementation of national strategies and technical guidelines together with national and local non-governmental organisations in a more ad-hoc fashion.

Participant identification and recruitment

Key child health stakeholders with country specific knowledge as well as non-health sector stakeholders on a national level in Cambodia were identified for participation by the research team. Participants were purposively selected based on predefined criteria of having expertise in child health or being from a non-health sector (for example water and sanitation, agriculture, infrastructure etc.) but with implementation knowledge of how child health interacts with other sectors in Cambodia. Efforts were made to recruit participants from many different sectors, including having participants from inside and outside of government. Further, the recruitment of participants was aimed to be balanced in terms of sex and seniority. The outreach to participants was done by DH, SS and TC through email and phone. The expected total number of participants was 30, balancing the need for reaching satisfactory information power[22] and feasibility.

Data collection

A total of 29 participants were interviewed between April-June 2020. Information was given verbally to all participants on the purpose of the study, what their involvement in the study would be, the risks

For peer review only

For peer review only - http://bmjopen.bmj.com/site/about/guidelines.xhtml

and benefits of taking part in the study, and that they had the right to decline participation or withdraw from the study at any time for any reason. Participants were asked to sign an informed consent form, written in Khmer before the interview started. The interviews were held in Khmer by authors SS and TC, audio recorded and transcribed verbatim into English. The interviews took place in Phnom Penh city vicinity, at the participant´s place of employment or other convenient but private location for the participant. An interview guide was developed based on established multisectoral frameworks; the SDG Synergies framework[27], Health in all policies approach[28] and multisectoral collaborative model presented by Kuruvilla et al[20] (see **Supplementary Material 1** for interview guide). The interview started with general background information on the participant, including the work experience in different sectors as represented by the Cambodian SDGs and moved on to the perception of the SDGs, child health and multisectoral collaboration and then focused on multisectoral collaboration for child health within the Cambodia context (identification of problem, design, implementation, and monitoring of the collaboration as well as relationships and capacity building activities). All types of collaborations between at least two or more sectors that had the explicit goal in some way to improve child health were considered during the interview. Two pilot interviews were held where after the interview guide was slightly adjusted for clarity.

Data analysis

Transcripts were imported into NVivo software for analysis. The transcripts were first analyzed by framework method analysis[29] by which the transcripts were read in full by DH, then coded through identification of meaning units, combining these into sub-categories and then grouped into overarching categories and lastly themes following the standard methodology. The themes, categories and sub-categories were inductively developed without prior anticipations [30] and continuously developed during the review of the transcripts. As such, the concepts of child health, SDGs and multisectoral collaboration emerged inductively. The coding was cross-checked by HMA and the analysis was continuously discussed with SS and TC to improve trustworthiness and validity.[22]

Patient and public involvement

No patients or public representative were directly involved in the design, conduct or reporting of this study. The findings will be disseminated and discussed with involved stakeholders. A reflexivity statement can be found in the **Supplementary Material 2.**

RESULTS

A diverse set of perspectives were provided by the participants (see **Table 1** for participant characteristics) on the research questions. Out of these, two main themes emerged in addition to several sub-themes and categories (**Table 2**, see **Supplementary Material 1** for full coding tables and COREQ checklist). The first theme related to the views of the participants on how the SDGs and expanded view on child health enable change the and the second main theme detailed the gap between theory and real-world complexities of conducting multisectoral collaborations for child health.

For peer review only - http://bmjopen.bmj.com/site/about/guidelines.xhtml

Table 1. Participant characteristics

Nr	Sex	Years worked	Organisation	Work sector experience according to Cambodian Sustainable Development Goals
1	Male	6-14	Governmental	7, 13, 14, 15
2	Female	>15	Governmental	3, 5, 6
3	Female	>15	Governmental	5, 17
4	Male	>15	Governmental	1, 3
5	Male	>15	Governmental	3, 4, 17
6	Female	1-5	Governmental	3, 16
7	Male	>15	Governmental	3, 4
8	Male	>15	Governmental	1, 2, 3, 4, 5, 6, 7, 10, 11, 12, 16, 17
9	Male	>15	Governmental	3, 4, 5, 16
10	Male	>15	Governmental	4, 17, 18
11	Male	>15	Governmental	4, 17, 18
12	Male	6-14	Governmental	1, 2, 3, 6, 16
13	Female	6-14	Governmental	1, 3, 17
14	Male	6-14	Governmental	17, 18
15	Male	6-14	Non-governmental	2, 3, 4, 6, 13

For peer review only - http://bmjopen.bmj.com/site/about/guidelines.xhtml

16	Male	>15	Non-governmental	2, 3
17	Male	>15	Non-governmental	5, 10, 16, 17
18	Male	6-14	Non-governmental	2, 3
19	Female	1-5	Non-governmental	1, 3, 4
20	Male	>15	Non-governmental	2, 4, 8
21	Male	>15	Non-governmental	16, 17
22	Female	>15	Non-governmental	2, 3, 4, 6
23	Male	>15	Non-governmental	3, 4, 5, 6
24	Female	>15	Non-governmental	2, 3, 5, 6
25	Male	>15	Non-governmental	1,2,3, 4, 6
26	Male	>15	Non-governmental	2, 3
27	Male	6-14	International	2, 3, 17
28	Female	6-14	International	1, 2, 3, 16, 17
29	Male	>15	International	2, 3, 4, 6, 17

Footnote: Cambodia Sustainable Development Goal 1 no poverty, 2 zero hunger, 3 child health, 4 quality education, 5 gender equality, 6 clean water and sanitation, 7 affordable and clean energy, 8 decent work and economic growth, 9 industry, innovation and infrastructure, 10 reduced inequalities, 11 sustainable cities and communities, 12 responsible consumption and production, 13 climate change, 14 life below water, 15 life on land, 16 peace, justice and strong institutions, and 18 mine/ERW free.

For peer review only - http://bmjopen.bmj.com/site/about/guidelines.xhtml

For peer review only

Table 2. Main themes, sub-themes and categories

**Themes**	SDGs and expanded view on child health enable change		Gap between theory and real-world complexities		
**Sub-themes**	Possibility for action due to SDGs	Higher ambitions for child health, a multisectoral area at heart	Planned linear process of collaboration	Real-world complexities shaping the collaboration	Critically assessing collaboration
**Categories**	SDGs provide a common vision and guide<br><br>Government commitment to and leadership of SDGs<br><br>Discrepancy between ambition and actual work	Definition of child health<br><br>Child health linkages across sectors<br><br>Aspects of the health system and actors unique to children<br><br>Special considerations for children	Identifying and framing problem<br><br>Actors and topics<br><br>Planning<br><br>Coordination<br><br>Implementation<br><br>Monitoring and evaluation<br><br>Dissemination	Funding<br><br>Relationships<br><br>Enabling environment<br><br>Capacity building	Success factors<br><br>Obstacles

For peer review only - http://bmjopen.bmj.com/site/about/guidelines.xhtml

SDGs and expanded view on child health enable change

Overall, interviewees reflected on the willingness by the national government to adopt the SDGs, how the possibility to achieving the SDGs depends on the outlook for the country while concluding that child health is a multisectoral topic at heart and that with the introduction of the SDGs the participants had set higher ambitions for child health and well-being.

Possibility for action due to SDGs

The 2030 Agenda and the SDGs were thought of as a universally relevant vision for sustainable development, providing a concrete roadmap or guide for each country. Comparing with the previous Millennium Development Goals, participants reflected on how the SDGs represent a more complex and detailed set of objectives that mirror actual conditions in the country. There was an overall agreement that the SDGs showcase that health is a multisectoral issue more clearly than during the Millennium Development Goals era. However, although the commitment to and leadership of the national government of Cambodia in adopting and implementing the Cambodian Sustainable Development Goals were evident, some participants noted the discrepancy between the highly set ambitions of the contextualised SDGs with the resources and work committed.

"That's the difference in perspectives between policymakers and implementers. The implementers in the ministry will complain about having lots of challenges and risks which could lead to a lower result. So, the plan to achieve many things by 2030 has already been written down. However, the implementation need budget and solutions to the challenge." - Nr 21, non-governmental organisation

Higher ambitions for child health, a multisectoral area at heart

Focusing on child health, most regarded children as people under the age of 18 and emphasised that physical and mental health are of equal importance to children. Interviewees detailed a range of linkages between child health and other sectors, mostly focusing on education and schooling, nutrition and other general societal conditions such as physical safety, environment, economic development and social protection systems. Overall, there was a strong notion of indivisibility between child health and its determinants, making the case that child health by definition is a multisectoral issue with all sectors responsible for its improvement.

"Like I mentioned, child health consists of physical, mental and social health. So, we need all relevant institutions to improve physical, mental and social health. We can't miss anyone to work on it." - Nr 5, governmental organisation

Interviewees put an emphasis on the family as responsible for the child´s health, while other stakeholders (government, international organisations and private sector) play an important role in shaping the determinants of child health in Cambodia. They further urged a concrete focus on preventive measures, improving quality and reach of health services related to the child and the family to improve child health further. Lastly, interviewees made the case for a life course approach to child health and setting a higher ambition for children with a focus on child growth and stronger acknowledgment of the rights of the child.

"To understand about the needs of children, we need to understand the growth of them first. Children's development consists of children before birth, children after birth to two years old, children in

For peer review only - http://bmjopen.bmj.com/site/about/guidelines.xhtml

kindergarten and primary school, and children in high school. The development of children on physical health, education and morality are ongoing process." - Nr 5, governmental organisation

Gap between theory and real-world complexities

When discussing multisectoral collaborations for child health, it became clear that there is a step-by-step linear process of thinking around the collaboration and its activities while aspects influencing the collaboration shape and direct the process in non-linear fashions. Participants also critically assess the collaborations, identifying success factors and obstacles for these types of collaborations in Cambodia.

Planned linear process of collaboration

The beginning of a multisectoral collaboration typically began with the identification and framing of a problem. This could be from a top-down approach, whereby government ministries identified a gap or need, or through policy or development plans while funding opportunities and the own organisational strategy or values could be other ways of identifying a problem. On the other hand, interviewees also described a bottom-up approach of problems being identified through routine data or findings from the grassroot level, complemented by listening and learning from community or sub-national stakeholders. The identified problem was often not primarily concerned with health but noted that child health might stand to benefit as an effect of a successful solution to the problem. The problem was typically framed in a detailed problem statement following involvement of many stakeholders in collective process, often using research in some way to narrow the problem.

"So, the needs can be identified through annual reports and through our observation in different sectors. Sometimes, we also do things following the donors' research and findings." – Nr 1, governmental organisation.

"They (government officials) collected all data from institutions under Ministry of Health. Then, they identified the challenge and priority action plans for next year. Besides, each unit need to monitor their annual results and to identify the priority action plans. That's how the Ministry of Health and different units identify the needs on child health, status, results and ways forward to reach SDGs."- Nr 6, governmental organisation.

The stakeholders involved in the discussed collaborations varied substantially, however the government (at national or sub-national level) was seen as a natural leader of collaborations while non-governmental organisations often organised in networks. Interviewees expressed territory feelings, with relatively strict boundaries between stakeholders and a critical view of government by the non-governmental organisations and vice versa.

"I am not blaming the government institutions, but there are some institutions which have too clear boundaries on their responsibilities and work. This leads to failure in our work. " – Nr 29, international organisation.

Planning of the collaboration were seen as a complex, detailed and resource demanding process. Often not formalised, a capacity assessment of the stakeholders in the collaboration, primarily focusing on

For peer review only - http://bmjopen.bmj.com/site/about/guidelines.xhtml

implementation capacity and not on specific knowledge or expertise in a particular sector or area were usually done at this stage, with the division of activities based on this assessment. If there was not enough implementation capacity to solve the problem identified, the collaboration could not begin. During the planning process interviewees noted that prioritization of activities was done depending on the funding requirements and to secure buy-in from certain stakeholders (particularly national government) seen as necessary for the success of the collaboration.

"For example, they (government servants) may plan 20 activities, but receive inadequate budget. So, they prioritize the activities to be done. According to my observation, district level is the same. They engage politics into their work. They like infrastructure development more than social development because it is eye-catching and visible." – Nr 9, governmental organisation.

Coordination was done in various ways depending on the collaboration however there were usually a common information sharing mechanisms, focal points at each stakeholder or joint committees with continuous coordination often built on somewhat already existing structures. There was also a clear division of responsibilities, although participation in joint coordination could be difficult to achieve and often those who coordinate do not have decision making power. Clear leadership of the collaboration was seen as paramount, with coordination succeeding or faltering based on the competence and willingness of the leader. As such, coordination was both a formal and informal process. Indeed, power and hierarchies shaped the coordination efforts where power imbalances or competition for funds between stakeholders could threaten the whole collaboration.

"Those people also need to have the authorization in decision-making in the meeting. In the past, there were people who attended the meeting, but did not do what were discussed. It was useless when people came to listen, but didn't share to their management and colleagues." – Nr 15, non-governmental organisation.

Implementation of the collaboration tried to follow the planning and set coordination mechanisms. However, collaborations were able to change depending on a change in the context or influencing aspects such as the Covid-19 pandemic or funding changes. Interviewees emphasised the difference between the national and sub-national level in terms of the collaboration, with larger collaborations having an administrative or policy function at the national level while implementation occurred at the sub-national level. This structure often led to increased complexities, with a different set of stakeholders needing to be involved at the different levels and the sub-national system having its own set of priorities.

"National level only work on policy. So, implementation goes to community level. I think that we should focus on provincial and communal level first to let them implement the work. We should also try to integrate the coordination with national level too by using forum to meet and discuss on the challenge." - Nr 28, international organisation.

Monitoring and evaluation were seen as integral to the collaboration, enabling learning and improvement of the collaboration itself and its activities and serving as the main accountability mechanism. The responsibility of conducting the monitoring and evaluation varied depending on the context and funding available, with external evaluation being seen as favourable if it could be funded. The national government and international organisations relied heavily on monitoring and evaluation for making decisions about the collaborations. However, it was seen as hard to move beyond pure

For peer review only - http://bmjopen.bmj.com/site/about/guidelines.xhtml

outputs, with quantitative indicators believed to be most reliable, and to attribute successes or failures to different stakeholders in the collaboration.

Dissemination of the collaboration and its activities were primarily thought of as information spreading, trying to raise awareness of the identified problem and engage the public and relevant stakeholders at national and international level in the efforts to solve it. It was also deemed important as a means of ensuring recognition from national level government ministries or the international community for the work done.

"We shared a lot, especially early childhood development program. We shared at provincial level and national committee on children education. We invited those committees to see our target location and our work. So, we disseminated a lot." Nr 22, non-governmental organisation.

Real-world complexities shaping the collaboration

There were a range of aspects influencing the process, often challenging the idea of a step-by-step linear approach of the collaboration. The most prominent aspect throughout was the funding, interviewees described the budget as the greatest limitation to the collaboration and called for more governmental funding at the national and sub-national level for multisectoral collaborations. Funding was seen as the most important source of power in the collaboration. Leadership roles, agenda setting and decision-making were mostly done by the organisation that controlled the funding.

"More importantly, we need the money to be available at sub-national level. The partners are all institutions. If the government can't manage to work on everything, we can ask civil society to help working on that. Nowadays, we are sceptical with NGOs. But, we also have example of government providing budget for NGOs to work." Nr 4, governmental organisation.

Relationships between the collaborators could facilitate or hamper the collaboration, with tensions between non-governmental organisations and the government existing and at the same time conflicts between government ministries or civil society networks that added complexity. For this reason, many collaborations tried to actively build relationships over time particularly between coordination focal points or joint committees, seeing mutual understanding leading to trust and confidence in the collaboration.

"The collaborative work also became better. During my time at education sectors, the relationship between partners was going very well, and we were happy to share any documents or data." – Nr 23, non-governmental organisation.

"There are many NGOs working to promote children. The government don't even know who they are. Some NGOs don't care about networking with the government too. This is the challenge according to my observation as a person in the middle of the two institutions. Both have their own weakness. Some NGOs do not know what the ministry have. For example, some NGOs do not know about existing guideline, plan or projects to work consistently. They only focus on their own work, and not pay attention to what others do to work collaboratively on the topic." – Nr 2, governmental organisation.

Capacity building were deemed to be key for the sustainability of the collaboration and its activities, particularly at the sub-national or implementation level, although demanding significant resources and the actual method varied depending on the type of collaborations and the stakeholders involved.

For peer review only

"Whenever there are requests from anyone or any organisations, we always respond and provide the training or sharing of experiences. We never hide our knowledge. We don't even charge them. We do it from our heart and soul. " – Nr 10, governmental organisation.

An enabling environment, particularly concerning policy and national governmental direction within which the collaboration took place, were seen as being of crucial importance. The introduction and adoption of the 2030 Agenda and the Cambodia Sustainable Development Goals, sub-national plans for development and national level plans promoted the idea of multisectoral collaboration. Government ministries that actively promoted or worked in multisectoral ways or through multisectoral committees, albeit not always successful, further promoted the advantages of tackling problems in a multisectoral fashion.

"The main thing is whether or not they have the commitment to work together. When commitment on that occur, the work can be done easily because visions created in country and global level has already been created." – Nr 17, non-governmental organisation.

Critically assessing collaborations

Interviewees reflected critically on their collaborations and had through experience identified some key success factors and often faced obstacles of multisectoral collaborations in Cambodia. Having clear responsibilities with agreement on division of activities, leadership from all and functioning monitoring and evaluation as well as a common vision and understanding based on continuous learning in an open environment where benefits and goals were explicit seem to be key success factors. Further, many emphasised the necessity of securing buy in, trust and commitment from all stakeholders in the collaboration from the beginning with the national government having a special role in all collaborations.

"Problems always occur. To work well with each other, we need to have collaborative plan with everyone's ownership. Secondly, we need to build trust and not allow any mistrust to happen." – Nr 27, international organisation.

"We also work closely and indirectly with selected institutions which have the most power." – Nr 3, governmental organisation.

Obstacles identified were lack of funding or long-term sustainability of the collaboration and its activities, with politics on sub-national and national level could mean unfavourable conditions for a collaboration or simply competing priorities or work of the stakeholders in the collaboration. There could also be a sense of a lack of accountability towards each other or the thought beneficiaries, with sometime faltering commitment to work together, lack of transparency of funds or efforts, and difficulty of attributing failures or successes.

"For instance if we are looking among 25 sub national civil society working group at the provincial/municipal level, there was only 50% who were active. Among these half, only 20 to 30 % who were very active in fulfilment of their collaborative work." – Nr 20, non-governmental organisation.

DISCUSSION

For peer review only - http://bmjopen.bmj.com/site/about/guidelines.xhtml

In this study, we found that the adoption of the SDGs led to an increased perceived possibility for action and higher ambitions for child health, perpetuating child health as a multisectoral issue. Further, there seem to be a gap between the desired step-by-step theory of conducting multisectoral collaboration and the real-world complexities of conducting such collaborations for child health in Cambodia. This is the first study to provide in-country insights that can be transferable on multisectoral collaborations for child health, overcoming some of the key methodological gaps noted by Glandon et al.[31] including describing power dynamics, type of governance arrangements and a diversity of stakeholder experiences.

The expanded view of child health and higher ambition for children to thrive led to a more compelling case for multisectoral collaborations to have a collaborative advantage over single-sector or single-stakeholder efforts. The 2030 Agenda and the SDGs influence social norms at a global, country, organisational and individual level.[32] The widespread knowledge of the overarching ambition and content of the SDGs in our study serve to exemplify the notion of universality of the 2030 Agenda, and the normative significance of universality in a country context.[33] Further, the perceived high ambition of the SDGs, the diversity of topics covered in the SDGs and their interlinked nature might shift norms to be more favourable towards multisectoral collaboration, in line with Huxam´s theory of collaboration advantage.[34] Placing children firmly in the centre of the SDGs in Cambodia might also allow for a re-vitalization of action and enable policy makers and practitioners to utilise the interlinkages within the SDGs to build multisectoral collaboration for child health.[35,36]

Multisectoral collaborations depicted by the participants in this study showcase that there is often no linear process but rather ongoing non-linear flow of activities that intentionally lead to a multisectoral collaboration (see **Supplementary Material 1** for illustrative examples of multisectoral collaborations). The rational logic of inquiry theory whereby one step lead to the next one until a decision is made and action is implemented and evaluated originally proposed by Dewey[37] were perceived by the participants to be the desired theory or process of collaboration. However as showcased by Kuruvilla and Dorstoewitz[38] previously, the collaborations described somewhat mimic the multisectoral collaboration model[20] which rests on dynamic networks and changing contexts. There was usually a capacity assessment of the potential or included stakeholders at the beginning of the collaboration, however it was usually described as informal or focused on securing funding and political buy-in rather than ensuring the implementation capacity of the collaboration, which could be why many collaborations had to divert from the desired linear process. Indeed, in our study participants singled out funding as an enabler and obstacle as well as a significant source of power in multisectoral collaborations. As noted by Rasanathan et al.[39] if multisectoral collaborations for health are to succeed appropriate financing systems that incentivise these collaborations must be in place, and the multisectoral monitoring and evaluation mechanisms allow for accountability. Conflicting perspectives between stakeholders, particularly government and non-governmental stakeholders, has been documented in Cambodia[25,26,40,41] and in other settings [42,43]. In our study there was a difference between interviewees from governmental organisations versus those from non-governmental particularly concerning the commitment and ability of the government to support and participate in multisectoral collaborations for child health. Although exploring this potential conflict was not the aim of this study, the emphasis of the participants on explicit and implicit territory feelings,

For peer review only - http://bmjopen.bmj.com/site/about/guidelines.xhtml

hierarchies and power dynamics at a national and sub-national level in Cambodia strengthen the need to include these concepts in collaborative theory and when designing multisectoral collaborations.[44,45]

Our limitations include that the purposive sampling led to selection bias in the recruitment of participants. As illustrated in Table 1, the interviewees were slightly unbalanced in terms of gender and work experience in SDG areas. Further, although much of the implementation of multisectoral collaborations is at the sub-national level the focus of this study was on the national level. Future studies might benefit from including participants with knowledge of collaborations on the sub-national level. Participants were asked to reflect on one or two multisectoral collaborations to inform the answers to the questions in the interview, they might have had a positive recall bias, only including those that were successful. Given the critical assessment of the multisectoral collaborations apparent in the interviews this seem negligible, however. Lastly, intra-personal dynamics between the interviewer and the interviewee might affect the answers and follow-up questions. In our study, the interviews were conducted by SS and TC, both representing academic institutions and being knowledgeable of qualitative research methods and the political landscape of organisations in Cambodia, ideally enabling both government and non-government stakeholders to express views and perceptions freely while adding credibility to the results. Although some of the findings in this study might reflect the unique Cambodia context, we believe that overall themes and conclusions are transferable to other middle-income countries and similar settings, adding valuable evidence on how stakeholders view multisectoral collaborations in general and specifically for child health. The study was designed to accomplish high information power across the five dimensions of information power,[22] however, with a broad research question and cross-case analysis the sample size was deemed to have to be relatively large to reach satisfactory information power and theoretical saturation. Information power was further increased by use of dense sampling method (purposive and specific), applied theory in the form of established frameworks for multisectoral collaborations, and high-quality dialogue in the interviews allowing for in-depth diverse multistakeholder perspectives.

CONCLUSION

We found that stakeholders in Cambodia perceived the SDGs to inspire an expanded view on child health that enabled change and promoted multisectoral collaboration. Interviewees experienced a gap between the desired theory of conducting multisectoral collaborations for child health and the real-world complexities of engaging in such an endeavour. The findings from this in-depth study can inform policy makers and practitioners who wish to encourage and take advantage of multisectoral collaborations for accelerating the work towards achieving better health in general and child health specifically the era of the SDGs.

Contributors

TA, HMA and DH conceived and designed the study. TC and SS contributed to the study design and conducted the data collection. DH analysed the data together with TC, SS and HMA. DH wrote the first

For peer review only - http://bmjopen.bmj.com/site/about/guidelines.xhtml

For peer review only

draft of the manuscript to which all authors (DH, TC, SS, HN SK, HMA and TA) provided critical contributions. All authors read and approved the final manuscript.

Funding

The work was supported by the Swedish Research Council (2018-03609). The funding organisation were not involved in the manuscript's writing or the decision to submit it for publication.

Competing interests

None declared.

Patient consent for publication

Not applicable.

Ethics approval

The study received ethical approval from the National Ethics Committee for Health Research in Cambodia (NECHR-023) and was exempt from ethical review from the Swedish Ethical Review Authority (Dnr 2022-00424-01). Written informed consent was obtained from all participants before inclusion in the study.

Data availability statement

No data is available. This is a qualitative study of a relatively small sample population in Cambodia. Making the dataset publicly available could potentially breach the privacy that was promised to participants when they agreed to take part and the ethical approvals granted. Therefore, the authors will not make the full transcripts available to a wider audience.

For peer review only

References

1 Independent Accountability Panel. Caught in the COIVD-19 storm: women' s , children' s , and adolescents ' health in the context of UHC and the SDGs. Geneva: 2020. https://iapewec.org/wp-content/uploads/2020/12/IAP-2020-Report_web-sp.pdf

2 United Nations. The Sustainable Development Goals Report. New York: 2022. https://unstats.un.org/sdgs/report/2022/The-Sustainable-Development-Goals-Report-2022.pdf

3 Marmot M. Achieving health equity: from root causes to fair outcomes. *Lancet* 2007;**370**:1153–63. doi:10.1016/S0140

4 Bishai DM, Cohen R, Alfonso YN, *et al.* Factors Contributing to Maternal and Child Mortality Reductions in 146 Low-and Middle- Income Countries between 1990 and 2010. *PLoS One* 2016;**11**:e0144908. doi:10.1371/journal.pone.0144908

5 Every Woman Every Child. The global strategy for women´s children´s and adolescents` health (2016-2030). New York: 2015.

6 Alfvén T, Dahlstrand J, Humphreys D, *et al.* Placing children at the center of the Sustainable Development Report: A SIGHT – Swedish Society of Medicine Road Map on Global Child

For peer review only - http://bmjopen.bmj.com/site/about/guidelines.xhtml

Health. Stockholm: 2019.

7 Lawn JE, Rohde J, Rifkin S, *et al.* Alma-Ata 30 years on: revolutionary, relevant, and time to revitalise. *Lancet (British Ed* 2008;**372**:917–27. doi:http://dx.doi.org/10.1016/S0140-6736(08)61402-6

8 Organization WH. Time to deliver: report of the WHO Independent High-Level Commission on Noncommunicable Diseases. Geneva: 2018.

9 Rasanathan K, Damji N, Atsbeha T, *et al.* Ensuring multisectoral action on the determinants of reproductive, maternal, newborn, child, and adolescent health in the post-2015 era. *BMJ* 2015;**351**:h4213. doi:10.1136/bmj.h4213

10 Levinson FJ, Balarajan Y, Marini A. Addressing malnutrition multisectorally. What have we learned from recent international experience? New York: 2013.

11 Lo S, Das P, Horton R. A good start in life will ensure a sustainable future for all. *Lancet* 2017;**389**:8–9. doi:10.1016/S0140-6736(16)31774-3

12 Neuman MJ, Devercelli AE. What matters most for early childhood development? A framework paper. Systems Approach for Better Education Results (SABER) working paper series. Washington: 2013.

13 Blomstedt Y, Bhutta ZA, Dahlstrand J, *et al.* Partnerships for child health: capitalising on links between the sustainable development goals. *BMJ* 2018;**360**:k125. doi:10.1136/BMJ.K125

14 Kuruvilla S, Schweitzer J, Bishai D, *et al.* Policy and Practice: Success factors for reducing maternal and child mortality. *Bull World Heal Organ* 2014;**92**:533–44. doi:10.2471/BLT.14.138131

15 Ahmed SM, Rawal LB, Chowdhury SA, *et al.* Cross-country analysis of strategies for achieving progress towards global goals for women's and children's health. *Bull World Health Organ* 2016;**94**:351–61. doi:10.2471/BLT.15.168450

16 UNICEF. Child Poverty in Cambodia Summary Report. Phnom Penh: 2018.

17 Kaba MW, Baesel K, Poch B, *et al.* IDPoor: a poverty identification programme that enables collaboration across sectors for maternal and child health in Cambodia. *BMJ* 2018;**363**:k4698. doi:10.1136/bmj.k4698

18 UNICEF. Reducing Stunting in Children Under Five Years of Age: A Comprehensive Evaluation of UNICEF's Strategies and Programme Performance – Cambodia Country Case Study. New York: 2017.

19 Royal Government of Cambodia. Cambodian Sustainable Development Goals (CSDGs) Framework (2016-2030). Phnom Penh: 2018.

20 Kuruvilla S, Hinton R, Boerma T, *et al.* Business not as usual: how multisectoral collaboration can promote transformative change for health and sustainable development. *BMJ* 2018;**363**:k4771. doi:10.1136/bmj.k4771

21 Tong A, Sainsbury P, Craig J. Consolidated criteria for reporting qualitative research: A 32-item checklist for interviews and focus groups. *Int J Qual Heal Care* 2018;**19**:349–57.

22 Malterud K, Siersma VD, Guassora AD. Sample Size in Qualitative Interview Studies: Guided by Information Power. *Qual Health Res* 2016;**26**:1753–60. doi:10.1177/1049732315617444

23 Bertelsmann Stiftung. BTI 2022 Country Report — Cambodia. 2022.

For peer review only

For peer review only - http://bmjopen.bmj.com/site/about/guidelines.xhtml

24 Royal Government of Cambodia. National Program for Sub-National Democratic Development. Phnom Penh: 2010.

25 Un K. *Cambodia: Return to Authoritarianism*. Cambridge University Press 2019.

26 Frewer T. Doing NGO Work: the politics of being ‘civil society’ and promoting ‘good governance’ in Cambodia. *Aust Geogr* 2013;**44**:97-114,. doi:10.1080/00049182.2013.765350

27 International Council for Science. A guide to SDG interactions: from science to implementation. Paris: 2017.

28 World Health Organization. Health in all policies: Helsinki statement. Framework for country action. Geneva: 2014.

29 Gale N, Heath G, Cameron E, *et al.* Using the framework method for the analysis of qualitative data in multi-disciplinary health research. *BMC Med Res Methodol 2013,* 2013;**13**:260–1.

30 Malterud K. Theory and interpretation in qualitative studies from general practice: Why and how? *Scand J Public Health* 2016;**44**:120–9. doi:10.1177/1403494815621181

31 Glandon D, Mondal S, Okeyo I, *et al.* Methodological gaps and opportunities for studying multisectoral collaboration for health in low- and middle-income countries. *Health Policy Plan* 2019;**34**:ii7–17. doi:10.1093/heapol/czz116

32 Schmieg G, Meyer E, Schrickel I, *et al.* Modeling normativity in sustainability: a comparison of the sustainable development goals, the Paris agreement, and the papal encyclical. *Sustain Sci* 2018;**13**:785–96. doi:10.1007/s11625-017-0504-7

33 Long G. The idea of universality in the sustainable development goals. *Ethics Int Aff* 2015;**29**:203–22. doi:10.1017/S0892679415000076

34 Huxham C. Theorizing collaboration practice. *Public Manag Rev* 2003;**5**:401–23. doi:10.1080/1471903032000146964

35 Alfvén T, Dahlstrand J, Humphreys D, *et al.* Placing children and adolescents at the centre of the Sustainable Development Goals will deliver for current and future generations. *Glob Health Action* 2019;**12**:1670015. doi:10.1080/16549716.2019.1670015

36 Clark H, Coll-seck AM, Banerjee A, *et al.* The Lancet Commissions A future for the world ’ s children ? A WHO – UNICEF – Lancet Commission. *Lancet* 2020;**395**:605–58. doi:10.1016/S0140-6736(19)32540-1

37 Dewey J. *Logic: The Theory of Inquiry*. New York: : Holt, Rinehart and Winston 1938.

38 Kuruvilla S, Dorstewitz P. There is no ‘point’ in decision-making: A model of transactive rationality for public policy and administration. *Policy Sci* 2010;**43**:263–87. doi:10.1007/s11077-009-9098-y

39 Rasanathan K, Atkins V, Mwansambo C, *et al.* Governing multisectoral action for health in low-income and middleincome countries: An agenda for the way forward. *BMJ Glob Heal* 2018;**3**:1–6. doi:10.1136/bmjgh-2018-000890

40 Schröder P, Young S. The Implications of Closing Civic Space for Sustainable Development in Cambodia. Sussex: 2019.

41 Norén-Nilsson A, Bourdier F. Introduction: Social Movements in Cambodia. *J Curr Southeast Asian Aff* 2019;**38**:3–9. doi:10.1177/1868103419848192

42 Rajabi M, Ebrahimi P, Aryankhesal A. Collaboration between the government and

For peer review only

For peer review only - http://bmjopen.bmj.com/site/about/guidelines.xhtml

nongovernmental organizations in providing health-care services: A systematic review of challenges. *J Educ Health Promot* 2021;**10**:1–9. doi:10.4103/jehp.jehp

43 Ejaz I, Shaikh B, Rizvi N. NGOs and government partnership for health systems strengthening:A qualitative study presenting viewpoints of government, NGOs and donors in Pakistan. *BMC Health Serv Res* 2011;**11**:122. doi:https://doi.org/10.1186/1472-6963-11-122

44 Faul M V. Multi-sectoral partnerships and power. Geneva: 2015.

45 Purdy JM. Power in Collaborative Governance. *Public Adm Rev* 2012;**72**:409–17. doi:10.111/j.1540-6210.2012.02525.x.A

For peer review only

For peer review only - http://bmjopen.bmj.com/site/about/guidelines.xhtml

Supplementary Material 1

Table of Contents

Interview Guide 2

Consolidated criteria for reporting qualitative studies (COREQ): 32-item checklist 6

Full coding tables 9

Examples of multisectoral collaboration that include child health and well-being in Cambodia 21

For peer review only

For peer review only - http://bmjopen.bmj.com/site/about/guidelines.xhtml

Interview Guide

1. Background information. *Please mark and fill in the following questions on this sheet*

1. How many years have you worked: ________

2. Sex ☐ Female ☐ Male

3. Education

Highest degree:____________________

Topic of degree:____________________

4. Work experience

Current employment

Organization:____________________ **Role/Position:**____________________

Work experience from the following sectors (represented by Cambodia Sustainable Development Goals) - Multiple Answers

Sectors	Rural	Urban	Sectors	Rural	Urban
1. Poverty/social protection	☐	☐	10. Income inequality	☐	☐
2. Food and nutrition	☐	☐	11. Sustainable cities/communities/urban planning	☐	☐
3. Health and well-being	☐	☐	12. Safe consumption and production	☐	☐
4. Education	☐	☐	13. Climate change	☐	☐
5. Gender equality	☐	☐	14. Life bellow water/ocean	☐	☐
6. Water and sanitation	☐	☐	15. Life on land/natural resources	☐	☐
7. Energy	☐	☐	16. Institutional strengthening/anti corruption/legislation	☐	☐
8. Labor market, financial sector	☐	☐	17. Partnerships/collaborative networks	☐	☐

For peer review only

For peer review only - http://bmjopen.bmj.com/site/about/guidelines.xhtml

9. Industry and infrastructure	☐	☐	18. Cambodia Mine/ERW free	☐	☐
Other:				☐	☐

Have you worked related to child health (both health and non-health sector)? ☐ Yes ☐ No

If yes, please describe shortly in what way:

Thank you for providing telling us your background, we would now like to know a little bit more about your views on the topics of Sustainable Development, child health and multisectoral work in general.

2. Background: Sustainable Development Goals, child health and multisectoral work

The 2030 Agenda and the 17 Sustainable Development Goals is a framework with a comprehensive set of goals adopted by the UN in 2015 for all countries to end poverty, protect the planet and ensure prosperity for all. These have been adapted to the Cambodia Sustainable Goals.

2.1. What were your first thoughts/opinion when the 2030 Agenda and the Sustainable Development Goals were launched?

2.1.2. What have your organization done now in relation to the Sustainable Development Goals in Cambodia compared to what you did with the Millennium Development Goals before and? (*mode of work rather than specific activities*)

2.2. Could you please describe what you would say child health includes?

2.2.1 What age? What to you include in the term "health" when it comes to children?

2.2.2 Are there any particular aspects of the health of children that you think the health system need to take special consideration to?

2.3. How do you think actors in Cambodia contribute in supporting child health to implement the Cambodia Sustainable Development Goals?

2.4. What are the linkages/connections between child health and non-health sectors (as represented by the Cambodia Sustainable Development Goals)?

2.4.1 Please provide examples of such linkages from your current/former work or what you have observe in the society?

2.4.2 Many sectors and activities can influence child health. Which sectors do you think are most relevant for child health?

For peer review only - http://bmjopen.bmj.com/site/about/guidelines.xhtml

Thank you for providing your views on these topics, now we would like to ask you some further questions on the multisectoral work around child health that you have experience from. Please think of a collaboration specifically, or generally if you have experience from many different, between two or more sectors that had the explicit goal to in some way increase child health and well-being.

3. Multisectoral collaboration for child health

Based on the Multisectoral collaborative model and the Health in all policies approach.

i) Drive change/Establishing the need for multisectoral work

3.1. Which organizations are your key stakeholders to work in promoting child health?

3.1. What was it that made the partners in the collaboration identify the need for multisectoral work? How did it begin?

ii) Defining the problem and constraints

3.2. How was the above-mentioned child health need identified, defined or framed?

iii) Design of the collaboration/Planned framed action & Supportive structures and policies

3.3. Was there a planning process of how to conduct the multisectoral work?

3.4. Which stakeholders were involved in planning?

3.5. How was the work of collaboration designed to be carried out?

3.5.1 How was the coordination organized?

3.5.2 How was the collaboration implemented?

3.5.3 How was the work of the collaboration financed or mobilized?

3.5. How do you think the multisectoral work were actually implemented compared to the plan?

3.6. Where there any supportive structures or policies in place that enabled the work to be conducted?

iv) Capture success / Monitoring and evaluation

3.7. How was the multisectoral work monitored and evaluated?

3.7.1 Was there any key indicators or markers of success monitored?

3.7.2 How was the success or failure attributed to between the partners in the collaboration?

V) Relate / Facilitate assessment and engagement & Build capacity

For peer review only - http://bmjopen.bmj.com/site/about/guidelines.xhtml

3.8. How did the relationship between the partners evolve during the multisectoral work?

3.9. Did the collaborating partners make any effort to improve their relationship?

3.10. Where there any efforts to engage with a wider group of actors or the public in the work?

4. Where there any type of capacity building activities included in the collaboration?

Final questions

5.1. What are your suggestions and recommendations in order to improve multisectoral collaboration to promote child health?

5.2. Are there any end points you want to add on any of the topics touched upon today or that we have not spoken about?

For peer review only

Consolidated criteria for reporting qualitative studies (COREQ): 32-item checklist

No.	Item	Description	Page
Domain 1: Research team and reflexivity			
Personal characteristics			
1	Interviewer/facilitator	SS and TC conducted the interviews	4
2	Credentials	DH has a MD, SS has PhD, TC has MD and has a Master of Arts in Health Social Sciences, HN has a PhD, KR has a PhD, HMA has a PhD and TA has a PhD.	1
3	Occupation	DH was a PhD candidate, SS was a lecturer at the Royal University of Phnom Penh, TC was a program manager at Malaria Consortium Cambodia.	1
4	Gender	All interviewers were male.	NA
5	Experience and training	The researchers (SS and TC) had extensive experience of qualitative interviews from previous research in Cambodia.	16
Relationship with participants			
6	Relationship established	No relationship was established prior to study commencement.	16
7	Participant knowledge of the interviewer	In some instances, the participant recognized the interviewer from attending similar events/workshops/seminars but in general the participants did not know the interviewers. They did not know the personal goals or reasons for doing the research for the individual interviewer.	16
8	Interviewer characteristics	The interviewers were all interested in the topic in general and had expertise in child health in Cambodia.	4
Domain 2: Study design			
Theoretical framework			

For peer review only - http://bmjopen.bmj.com/site/about/guidelines.xhtml

9	Methodological orientation and theory	The methodological orientation of the study is content analysis, specifically framework analysis.	4
Participant selection			
10	Sampling	Participants were purposively selected based on predefined criteria of having expertise in child health or being from a non-health sector (for example water and sanitation, agriculture, infrastructure etc.) but with implementation knowledge of how that sector interacts with other sectors in Cambodia.	4
11	Method of approach	Participants were approached via email and telephone.	NA
12	Sample size	29 participated in the interviews.	4
13	Non-participation	No participants refused or dropped out.	4
Setting			
14	Setting of data collection	The interviews took place either virtually (over online meeting) or face to face, at a time and place convenient of the participant.	4
15	Presence of non-participants	The were no non-participants present during the interviews.	4
16	Description of sample	The description of the sample can be seen in Table 1 in the article.	5
Data collection			
17	Interview guide	This is provided in the supplementary material 1. The interview guide was piloted before the study began.	4-5
18	Repeat inverviews	No repeat interviews were held.	NA
19	Audio/visual recording	Audio recording was used to collect the data.	4
20	Field notes	No field notes were taken.	4
21	Duration	The duration of the interviews ranged from 45 minutes to 1 hr and 15 minutes	NA
22	Data saturation	Is discussed with regards to information power in the article.	16

For peer review only - http://bmjopen.bmj.com/site/about/guidelines.xhtml

23	Transcripts returned	Transcripts were not returned to participants.	NA
Domain 3: Analysis and findings			
Data analysis			
24	Number of data coders	DH coded the data	8
25	Description of the coding tree	Is presented in Table 2 in the manuscript and supplementary material 1.	9
26	Derivation of themes	The themes were derived from the data.	8
27	Software	Nvivo software were used for the coding.	8
28	Participant checking	The participants did not provide feedback on the findings.	NA
Reporting			
29	Quotations presented	Quotations presented with each paragraph, trying to illustrate the main points.	10-14
30	Data and findings consistent	The data and findings were cross-checked multiple times, ensuring consistency.	16
31	Clarity of major themes	Outlined in result table and in clear headings in the result section.	9-14
32	Clarity of minor themes	Outlined in result table and in clear headings in the result section.	9-14

For peer review only

Full coding tables

Main themes and findings - full coding tables

**Theme**	SDGs and expanded view on child health enable change			
**Sub-themes**	Possibility for action due to SDGs			
**Categories**	Government commitment to and leadership of SDGs	SDGs provide a common vision and guide		Discrepancy between ambition and actual work
**Subcategories**		More detailed than MDGs	Showcase that health is a multisectoral issue	
**Codes**	Adoption and change of national plans and policies<br><br>No change in government as leaders of the goals<br><br>SDG implementation depends on alignment to government	Provide a clear set of goals<br><br>Provide a roadmap or guide<br><br>More detailed<br><br>More complex reflecting actual conditions	Illustrate that health is a multisectoral issue<br><br>SDGs reflecting actual conditions with regards to health	SDGs too complex, impossible to succeed<br><br>High ambition not matched with resources/work committed

For peer review only

For peer review only - http://bmjopen.bmj.com/site/about/guidelines.xhtml

Main themes and findings - full coding tables, continued.

**Themes**	SDGs and expanded view on child health enable change					
**Sub-themes**	Higher ambitions for child health, a multisectoral area at heart					
**Categories**	Definition of child health		Child health linkages across sectors			
**Sub-categories**	Child age under 18 years	A focus on not only health but well-being	Child health by definition a multisectoral issue	Education and schooling	Nutrition	General societal conditions
**Codes**	General view and legally a child is a person under 18 years of age	Physical and mental health equally important<br><br>Good nutrition and absence of disease	All SDGs important for child health<br><br>The linkages between sectors and child health cannot be divided	Education as most formative experience<br><br>School important physical place for linkages<br><br>Early child development key	Nutrition and functioning agricultural sector as basis for child growth<br><br>Commercial interests conflicts with good child nutrition	Physical safety and hygiene environment<br><br>Economic development of country<br><br>Social protection systems

For peer review only

Main themes and findings - full coding tables, continued.

**Theme**	SDGs and expanded view on child health enable change				
**Sub-themes**	Higher ambitions for child health, a multisectoral area at heart				
**Categories**	Aspects of the health system and actors unique to children			Special considerations for children	
**Sub-categories**	Responsibility of family and community	Influence of other actors	Key aspects of health system for improving child health	Life course approach	Enabling the child to thrive
**Codes**	Parents and family are the primary caretaker<br><br>Information and health literacy key undertaking<br><br>Social determinants of family dictates child health to large extent	Government overarching leader and supporter of child health<br><br>International organizations influence organizations in country<br><br>Commercial interests of private sector	Lack of focus on preventive child health measures<br><br>Need to improve quality and equity<br><br>Difference between rural and urban areas	Prenatal services important for child health<br><br>Children have different needs at different ages	A focus on child growth<br><br>Holistic approach<br><br>Acknowledging child rights

For peer review only - http://bmjopen.bmj.com/site/about/guidelines.xhtml

Main themes and findings - full coding tables, continued.

**Theme**	Gap between theory and real world complexities					
**Sub-themes**	Linear process of collaboration					
**Categories**	Actors and topics			Identifying and framing problem		
**Sub-categories**	Broad variety of actors	Territory feelings	Collaborations focused on non-health aspects	Top-down approach	Bottom-up approach	Framing of problem
**Codes**	Government as natural leader<br><br>Civil society networks<br><br>External donors emphasize importance<br><br>Many different actors collaborating	There exist strict boundaries between actors<br><br>Competition between actors for funding<br><br>Skeptical view of government and NGO and vice versa	Focused on preventive issues<br><br>Collaboration indirectly see effect on child health<br><br>Willingness to connect to child health	Government or ministries identifies need<br><br>National policy or development plan<br><br>International agenda or external funding opportunities<br><br>From own organizational strategy or values	Listening to stakeholders in community or on sub-national level<br><br>Routine data or findings from actual situation on the ground<br><br>Reliable data not always present	Research as a way of narrowing problem<br><br>Involving many actors in collective process<br><br>Detailed problem statement

For peer review only - http://bmjopen.bmj.com/site/about/guidelines.xhtml

Main themes and findings - full coding tables, continued.

**Theme**	Gap between theory and real world complexities						
**Sub-themes**	Linear process of collaboration						
**Categories**	Planning			Coordination			
**Sub-categories**	Complex, detailed, resource constraining process	Capacity assessment key	Prioritization depending on context	Varied methods of coordination	Clear division of responsibilities	Leadership paramount	Power and hierarchies influence coordination
**Codes**	Many actors involved in planning<br><br>Sub-national and national level engaged<br><br>Technical level and strategy level<br><br>Detailed collaboration plan and outline of activities, outputs,	Technical skill and resource capacity at implementer level instrumental<br><br>Division of activities based on capacity<br><br>If not enough capacity collaboration cannot begin	Prioritization based on funding requirements<br><br>Politics and benefits of including certain actors or activities	Information sharing mechanisms<br><br>Focal points or joint committees<br><br>Regular, continuous coordination<br><br>Built on existing structures	Agreed upon plan of responsibilities<br><br>Common vision and commitment key for ease of coordination<br><br>Participation in joint coordination hard	Single organization that explicitly or implicitly lead<br><br>Structuring collaboration efforts depends on leader	Focal points for collaboration lack decision making power<br><br>Power imbalance due to government more powerful<br><br>Competing for funding between organizations

For peer review only - http://bmjopen.bmj.com/site/about/guidelines.xhtml

	and desired outcomes<br><br>Commitment and ownership implicit goals of process						

For peer review only - http://bmjopen.bmj.com/site/about/guidelines.xhtml

Main themes and findings - full coding tables, continued.

**Theme**	Gap between theory and real world complexities						
**Sub-themes**	Linear process of collaboration						
**Categories**	Implementation			Monitoring and evaluation			
**Sub-categories**	Adaptability to change	Geographical and administrative level	Follows from planning and coordination	Detailed but depends on funding	Hard to move beyond outputs	Integral to the collaboration	Responsibility for M&E varies
**Codes**	Implementation does not follow plan<br><br>Funding changes requires change of plan<br><br>Government involvement lead to less flexibility<br><br>Covid-19 disruption	Focus on implementing organizations or participations<br><br>Added complexity for actual implementation<br><br>National level collaboration, sub-national implement<br><br>Sub-national own system of priorities,	Implementation mirrors previous collaborative efforts<br><br>Reduction in parallel work and efficient implementation<br><br>Takes time and resources to implement, need to be considered before start	Funding source and resources key for allowing M&E<br><br>M&E include detailed indicators<br><br>Government or external donor relies heavily on M&E for decisions	Discrepancy in M&E between stakeholders<br><br>Particularly hard to attribute success or failures<br><br>Quantitative indicators more favorable	Learning from failure<br><br>M&E seen as opportunity to learn and improve<br><br>Successes can build momentum, secure resources<br><br>Serves as main accountability mechanism	Internal or external evaluation depending on context and resources<br><br>One stakeholder monitors activities<br><br>Joint monitoring of activities

For peer review only - http://bmjopen.bmj.com/site/about/guidelines.xhtml

		relationships and focus					

For peer review only - http://bmjopen.bmj.com/site/about/guidelines.xhtml

Main themes and findings - full coding tables, continued.

**Theme**	Gap between theory and real world complexities						
**Sub-themes**	Linear process of collaboration		Real-world complexities shaping the collaboration				
**Categories**	Dissemination		Funding		Relationships		
**Sub-categories**	Information spreading	Recognition	Call for more funding	Funding as a source of power	Facilitate or hamper collaboration	Actively building relationships	Relationships as an outcome
**Codes**	Engage the public and stakeholders<br><br>Increase awareness	Engaging national-level government<br><br>Gain international reputation	Budget greatest limitation to collaboration<br><br>Not enough government/national funding<br><br>Funding sources varies<br><br>If government funding more sustainable	External donors agenda decide activities<br><br>If funding from government they have last say<br><br>Leadership often based on funding<br><br>Ministry of economy key stakeholder	Tensions between NGOs and government evident<br><br>Conflicts within government or NGO networks<br><br>Common understanding and relationships increase coordination	Continuous relationship building<br><br>Efforts by stakeholders to build relationships	Over time relationships built through coordination meetings and implementation<br><br>Evolve between key focal points<br><br>Mutual understanding lead to trust and confidence

For peer review only

For peer review only - http://bmjopen.bmj.com/site/about/guidelines.xhtml

				Decide design and coordination of collaboration			

For peer review only - http://bmjopen.bmj.com/site/about/guidelines.xhtml

Main themes and findings - full coding tables, continued.

**Theme**	Gap between theory and real world complexities									
**Sub-themes**	Real-world complexities shaping the collaboration					Critically assessing collaborations				
**Categories**	Enabling environment		Capacity building			Success factors			Obstacles	
**Sub-categories**	Policies	Government	Actual method depends on collaboration	Key for sustainability	Demands resources	Clear responsibilities	Common vision and understanding	Secure buy in	Real world complexities	Lack of accountability
**Codes**	International agenda facilitate work<br><br>Sub-national plans for development<br><br>National CSDG roadmap and other national plans	Active and collaborative government ministries<br><br>Existing multisectoral ministerial committees	In person technical capacity building<br><br>Natural reciprocal<br><br>Effort to include capacity building<br><br>Capacity building according to administrative	Learning and incorporating changes<br><br>Integral part of collaboration itself, one of main benefits<br><br>Building capacity with implementors or sub/national level lead to sustainability	Capacity building takes time<br><br>Capacity building limited by funding	Agreement on division of activities<br><br>Leadership from all<br><br>Functioning M&E	Learning continuously<br><br>Open sharing and discussion<br><br>Benefits and goals explicit	Engage stakeholders from beginning<br><br>Government and sustainable funding<br><br>Commitment from all<br><br>Relationship and capacity building	Lack of funding, sustainability<br><br>Politics on sub-national and national level<br><br>Competing priorities and work	No commitment to work together<br><br>Lack of transparency<br><br>Difficulty of attributing failures or successes

For peer review only - http://bmjopen.bmj.com/site/about/guidelines.xhtml

			and geographical level							

For peer review only - http://bmjopen.bmj.com/site/about/guidelines.xhtml

Examples of multisectoral collaboration that include child health and well-being in Cambodia

Name	Short description	Source
Multisectoral Food and Nutrition Security in Cambodia (MUSEFO)	Through a multisectoral approach, the programme aims to improve the nutrition of women and young children through i) Improving the quality of nutrition services by providing training for health workers. ii) Diversifying nutrition and food production by providing trainings for farmers, building their capacity to grow a more diverse range of crops and improving their access to healthy foods. iii) Embedding successful approaches on national and regional level is the third field of action.	https://giz-cambodia.com/wordpress/wp-content/uploads/10_FactSheet-of-Multisectoral-Food-and-Nutrition-Security-in-Cambodia-MUSEFO.pdf
Identification of Poor Households Programme (ID Poor)	The ID Poor program aim to identify at risk or poor households in Cambodia and provide Equity Cards to these households as a basis for assessing social assistance services. This can then be used by various ministries or other organizations to assist at-risk households with healthcare for children.	https://idpoor.gov.kh/en/
The Second National Strategy for Food Security and Nutrition 2019-2023	Acknowledging the cross-cutting challenges facing the ambition to provide proper food and nutrition, including promoting infant breastfeeding practices, the government has implemented a national-wide strategy which explicitly take an multisectoral approach to nutrition.	https://scalingupnutrition.org/sites/default/files/2022-06/national-nutrition-plan-cambodia.pdf
Raising Awareness and Innovative	Led by Save the Children, the project used innovative approaches to increase awareness and appreciation of a	https://resourcecentre.savethechildren.net/pdf/RAISE-Evaluation-Report.-Final.-16-March-2022-1.pdf/

For peer review only - http://bmjopen.bmj.com/site/about/guidelines.xhtml

Strategies for ECD (RAISE)	holistic approach to early childhood development in 43 villages in Kampong Siem district.	
Family Care First (FCF) and Responsive and Effective Child Welfare Systems Transformation (REACT)	Facilitated by Save the Children, the project is a multi-donor supported network of organizations including government, NGO´s and UN organizations working together to support children to live in safe, nurturing family-based care. The work take place across numerous sectors and stakeholders.	https://resourcecentre.savethechildren.net/pdf/ Gender%20Intersectionality%2 0and%20 Family%20Separation%20Alternative%20Care%20and%20%20the%20 Reintegration%20of20Children%20FINAL_0.pdf/
The Fifth National Strategic Plan for a Comprehensive, Multi-Sectoral Response to HIV/AIDS (2019-2023)	Through a multisectoral approach and partnership across ministries and different organizations, Cambodia is working towards the 90-90-90 targets and eventually elimination of new HIV infections including mother-to-child transmission.	http://www.healthpolicyplus.com/ns/pubs/17402-17725_CambodiaStrategicPlan.pdf

For peer review only

For peer review only - http://bmjopen.bmj.com/site/about/guidelines.xhtml

Supplementary Material 2. Reflexivity Statement

Study conceptualisation:

1. How does this study address local research and policy priorities?

This study is a part of an effort to provide country specific knowledge of multisectoral collaborations in Cambodia, a key knowledge gap identified by stakeholders and academia. The government and other organizations are actively engaging in multisectoral collaborations, and understanding how the function in practice is a key priority.

2. How were local researchers involved in study design?

The local researchers (SS and TC) were engaged in the overall design of the study and particularly the identification and recruitment of participants as well as development of the interview guides and data collection. They were core members of the study team.

Research management:

1. How has funding been used to support the local research team(s)?

The study was funded through the Swedish Research Council (2018-03609) with the majority of funding dedicated to country study activities and local research colleagues (SS and TC).

Data acquisition and analysis:

1. How are research staff who conducted data collection acknowledged?

The researchers who conducted data collection met the authorship criteria and are hence acknowledged as co-authors of the study.

2. How have members of the research partnership been provided with access to study data?

All members of the research team, including SS and TC, had full access to the data.

3. How were data used to develop analytical skills within the partnership?

The qualitative data analysis was conducted by DH with input and training of DH, SS and TC by a qualitative research expert (HMA).

Data interpretation:

1. How have research partners collaborated in interpreting study data?

The results from the study were continuously discussed with the local research colleagues (SS and TC) who contributed significantly to the interpretation of the results.

Drafting and revising for intellectual content:

1. How were research partners supported to develop writing skills?

Most of the writing of the manuscript was done by DH, however local research colleagues (SS and TC) provided crucial input.

For peer review only

2. How will research products be shared to address local needs?
The results from the study will be disseminated widely to an international and national audience, including a dissemination seminar with relevant country stakeholders.

Authorship:

1. How is the leadership, contribution and ownership of this work by LMIC researchers recognized within the authorship?
The local researchers (SS and TC) authors 2-3, recognizing their crucial hands-on contribution to the study.

2. How have early career researchers across the partnership been included within the authorship team?
The first author is a PhD student (although not from a LMIC), SS and TC are recognized experienced researchers.

3. How has gender balance been addressed within the authorship?
Out of the seven authors, four are male (DH, SS, TC and TA) while three (HN, SK and HMA) are female. The preponderance for male authors is weighted against the critical study design and interpretation by HN and SK while HMA is a world-leading qualitative expert.

Training:

1. How has the project contributed to training of LMIC researchers?
The LMIC researchers (SS and TC) are experienced qualitative researchers, however within this study all authors gained refresher trainings and developed their qualitative analytical skills and knowledge of framework method analysis by HMA (qualitative expert).

Infrastructure:

1. How has the project contributed to improvements in local infrastructure?
No direct benefit in local infrastructure has come from this qualitative study, however the findings of the study can help to conceptualize and form partnerships across sectors that can lead to improvements in infrastructure.

Governance:
1. What safeguarding procedures were used to protect local study participants and researchers?
The study conforms to the Helsinki declaration and followed the ethical and practical guidelines stipulated by the National Ethics Committee for Health Research in Cambodia regarding the safety of researchers and participants.

For peer review only

Sustainable development goals and multisectoral collaborations for child health in Cambodia: a qualitative interview study with key child health stakeholders

Consolidated criteria for reporting qualitative studies (COREQ): 32-item checklist

No.	Item	Description	Page
Domain 1: Research team and reflexivity			
Personal characteristics			
1	Interviewer/facilitator	SS and TC conducted the interviews	4
2	Credentials	DH has a MD, SS has PhD, TC has MD and has a Master of Arts in Health Social Sciences, HN has a PhD, KR has a PhD, HMA has a PhD and TA has a PhD.	1
3	Occupation	DH was a PhD candidate, SS was a lecturer at the Royal University of Phnom Penh, TC was a program manager at Malaria Consortium Cambodia.	1
4	Gender	All interviewers were male.	NA
5	Experience and training	The researchers (SS and TC) had extensive experience of qualitative interviews from previous research in Cambodia.	16
Relationship with participants			
6	Relationship established	No relationship was established prior to study commencement.	16

For peer review only - http://bmjopen.bmj.com/site/about/guidelines.xhtml

7	Participant knowledge of the interviewer	In some instances, the participant recognized the interviewer from attending similar events/workshops/seminars but in general the participants did not know the interviewers. They did not know the personal goals or reasons for doing the research for the individual interviewer.	16
8	Interviewer characteristics	The interviewers were all interested in the topic in general and had expertise in child health in Cambodia.	4
Domain 2: Study design			
Theoretical framework			
9	Methodological orientation and theory	The methodological orientation of the study is content analysis, specifically framework analysis.	4
Participant selection			
10	Sampling	Participants were purposively selected based on predefined criteria of having expertise in child health or being from a non-health sector (for example water and sanitation, agriculture, infrastructure etc.) but with implementation knowledge of how that sector interacts with other sectors in Cambodia.	4
11	Method of approach	Participants were approached via email and telephone.	NA
12	Sample size	29 participated in the interviews.	4
13	Non-participation	No participants refused or dropped out.	4
Setting			
14	Setting of data collection	The interviews took place either virtually (over online meeting) or face to face, at a time and place convenient of the participant.	4
15	Presence of non-participants	The were no non-participants present during the interviews.	4
16	Description of sample	The description of the sample can be seen in Table 1 in the article.	5
Data collection			

For peer review only - http://bmjopen.bmj.com/site/about/guidelines.xhtml

17	Interview guide	This is provided in the supplementary material 1. The interview guide was piloted before the study began.	4-5
18	Repeat inverviews	No repeat interviews were held.	NA
19	Audio/visual recording	Audio recording was used to collect the data.	4
20	Field notes	No field notes were taken.	4
21	Duration	The duration of the interviews ranged from 45 minutes to 1 hr and 15 minutes	NA
22	Data saturation	Is discussed with regards to information power in the article.	16
23	Transcripts returned	Transcripts were not returned to participants.	NA
Domain 3: Analysis and findings			
Data analysis			
24	Number of data coders	DH coded the data	8
25	Description of the coding tree	Is presented in Table 2 in the manuscript and supplementary material 1.	9
26	Derivation of themes	The themes were derived from the data.	8
27	Software	Nvivo software were used for the coding.	8
28	Participant checking	The participants did not provide feedback on the findings.	NA
Reporting			
29	Quotations presented	Quotations presented with each paragraph, trying to illustrate the main points.	10-14
30	Data and findings consistent	The data and findings were cross-checked multiple times, ensuring consistency.	16
31	Clarity of major themes	Outlined in result table and in clear headings in the result section.	9-14
32	Clarity of minor themes	Outlined in result table and in clear headings in the result section.	9-14

For peer review only - http://bmjopen.bmj.com/site/about/guidelines.xhtml
